# Rifamycin Structural Modifications Attenuate PXR Binding and CYP3A4 Induction

**DOI:** 10.1021/acs.jmedchem.6c00944

**Published:** 2026-06-23

**Authors:** Amir George, Tian Lan, Andrew D. Huber, Véronique Dartois, Thomas Dick, Taosheng Chen, Courtney C. Aldrich, Joel S. Freundlich

**Affiliations:** † Department of Pharmacology, Physiology, and Neuroscience, Rutgers University—New Jersey Medical School, Newark, New Jersey 07103, United States; ‡ Department of Medicinal Chemistry, University of Minnesota, Minneapolis, Minnesota 55455, United States; § Department of Chemical Biology and Therapeutics, St. Jude Children’s Research Hospital, Memphis, Tennessee 38105, United States; ∥ Center for Discovery & Innovation, 3139 Hackensack Meridian Health, Nutley, New Jersey 07110, United States; ⊥ Department of Medical Sciences, Hackensack Meridian School of Medicine, Nutley, New Jersey 07110, United States; # Department of Microbiology and Immunology, Georgetown University, Washington, District of Columbia 20057, United States; ¶ Division of Infectious Disease, Department of Medicine and the Ruy V. Lourenço Center for the Study of Emerging and Re-emerging Pathogens, Rutgers University—New Jersey Medical School, Newark, New Jersey 07103, United States

## Abstract

Rifamycins are a cornerstone of antimycobacterial therapy. However, their clinical use is limited by drug–drug interactions, arising from activation of the nuclear receptor pregnane X receptor (PXR). PXR activation induces expression of drug-metabolizing enzymes, including the cytochrome P450 3A4 isoform (CYP3A4), and accelerates clearance of coadministered medications. The structural understanding of rifamycin–PXR interactions remains limited. We designed a series of C25-modified rifabutin analogs and systematically evaluated their PXR binding, transcriptional activation, and pharmacological profile. Several analogs retained PXR binding affinity yet showed reduced CYP3A4 induction, exhibiting behavior consistent with antagonists or inverse agonists and revealing a disconnect between receptor binding and transcriptional activation. Molecular dynamics simulations indicated C25 modifications may disrupt positioning of the PXR α12 helix through steric interactions. These findings demonstrate conservative modifications of rifamycin can potentially convert PXR agonists into antagonists or inverse agonists and offer a structure-guided framework for developing rifamycins with attenuated CYP3A4 induction.

## Graphical abstract

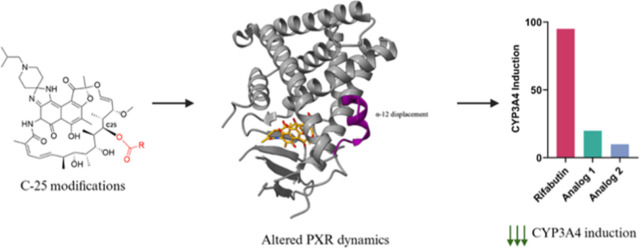

## Introduction

Rifamycin drugs have long been considered one of the main pillars of the treatment of mycobacterial infections caused by *Mycobacterium tuberculosis* and pathogenic nontuberculous mycobacteria (NTM). The antibiotic rifampicin (Figure 1A) is a first-line antitubercular drug, and its use in combination with isoniazid, pyrazinamide, and ethambutol was associated with an 88% global treatment success for drug-susceptible tuberculosis (TB) globally in 2023.^1^ However, a major drawback of rifampicin and other rifamycin class drugs, including rifapentine and rifabutin, is their significant drug–drug interactions (DDIs) with a wide variety of companion drugs, whose exposure and hence efficacy are substantially decreased upon coadministration.^2^ For example, rifampicin pretreatment reduces the exposure of midazolam by up to 98%,^3^ simvastatin by up to 87%,^4^ and saquinavir by up to 70%^5^ in healthy volunteers. Since rifamycin drugs are prescribed in multidrug regimens for patients suffering from mycobacterial infections and often other comorbidities/coinfections (e.g., HIV-1, cystic fibrosis), DDIs pose a particular challenge and seriously limit further application of these foundational antibacterials.

**Figure 1:**
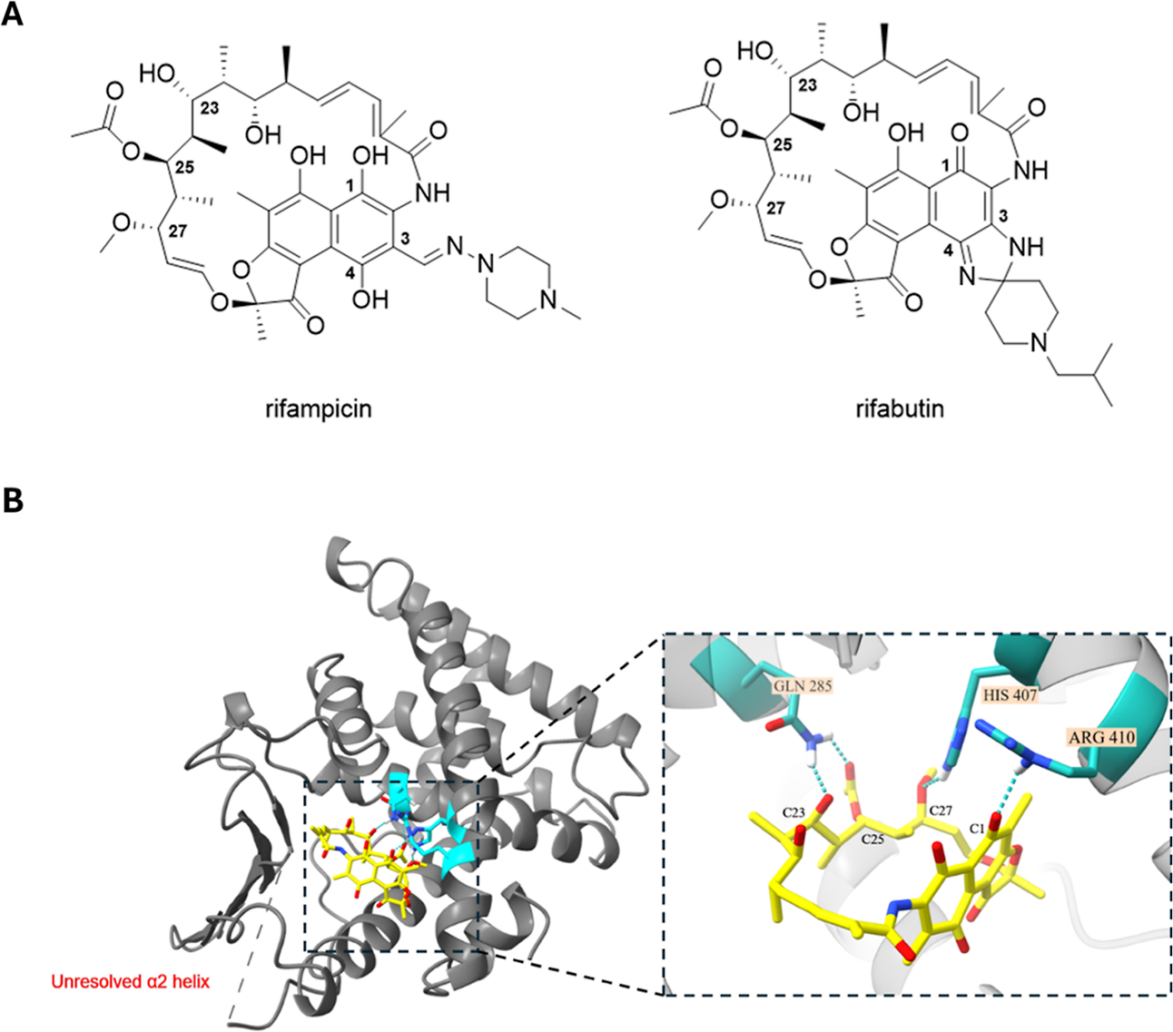
(A) Chemical structures of rifampicin and rifabutin. Relevant numbering is shown. (B) X-ray crystal structure of the PXR LBD (gray) in complex with rifampicin (yellow) (PDB ID: 1SKX). Residues 178–209, which comprise the α2 helix of PXR adjacent to rifampicin, are disordered and exhibit no clear electron density. The 4-methylpiperazine ring of rifampicin is likewise not resolved in the electron density. Hydrogen bonds between PXR residues Gln285, His407, and Arg410 (cyan) and the rifampicin ansa chain are shown with cyan dashed lines. This figure was created with UCSF ChimeraX (Version 1.11).

Rifamycin DDIs largely stem from the induction of drug-metabolizing enzymes, especially cytochrome P450 (CYP) enzymes that account for the metabolism of about 75% of current drugs.^6^ Rifamycins induce CYPs, particularly the most abundant isoform CYP3A4, by binding to the ligand-activated nuclear receptor pregnane X receptor (PXR).^7–9^ PXR acts as a master sensor of xenobiotics and promotes their elimination via escalating certain detoxification pathways such as metabolism and efflux.^10^ Rifamycins bind the PXR ligand-binding domain (LBD) in either the monomeric state or the heterodimer formed with retinoid X receptor (RXR). Ligand binding promotes dissociation of corepressors and recruitment of coactivators, thereby activating transcription at response elements of target genes and upregulating their expression.^11^ We hypothesize that alteration of the rifamycin structure to reduce or eliminate PXR activation may provide a promising strategy to reduce DDIs and further strengthen the clinical value of rifamycin antibacterials.

To test this hypothesis, we began with the inspection of the rifampicin-PXR LBD X-ray crystal structure.^12^ In this X-ray crystal structure, the *N*-methyl piperazinyl hydrazone tail substituting the rifampicin C3 position appeared flexible and lacked electron density, while the remaining structure was clearly resolved in the binding pocket. Particularly, the aliphatic ansa-chain moiety of rifampicin formed multiple close interactions with residues of the binding pocket including four hydrogen bonds with the C1-OH, C23-OH, C25-OAc and C27-OMe moieties (Figure 1B).^12^ Although the majority of reported rifamycin optimization efforts to lower CYP induction have focused on the substituents of the C3 and C4 positions,^13,14^ we postulated that structural modification of the rifamycin ansa-chain would more efficiently alter the binding with PXR and consequently attenuate CYP3A4 induction. Herein, we describe our pursuit of ansa-chain-modified rifamycin analogs with significant attenuation of CYP3A4 induction using structure-based design strategies. Our investigations into in vitro PXR LBD binding properties and cellular PXR-mediated response as a function of computationally predicted PXR interactions suggest novel molecular mechanisms for rifamycin modulation of CYP3A4 induction. Given the central role of rifamycins in treating mycobacterial infections and the dosing constraints imposed by their PXR-mediated DDIs, our findings provide a conceptual framework for designing next-generation rifamycins with attenuated PXR activation and CYP3A4 induction, thereby expanding their safe use in combination therapy.

## Results

### Ansa-Chain Modification Enables Attenuation of CYP Induction

Given that the rifamycin ansa-chain directly interacts with PXR residues buried deep within the binding pocket, we postulated that a bulky modification on the ansa-chain would similarly disrupt PXR binding. We chose to investigate modification of the C25 position on the ansa-chain since previous C25 modifications were demonstrated to not affect the antibacterial activity.^15,16^ One of the major mechanisms of resistance to rifampicin in mycobacteria is ADP-ribosylation^17^ and C25 modifications have been shown to preclude intracellular ADP-ribosylation in mycobacteria.^18,19^ Furthermore, bulky substituents at the 3-position of rifamycins have been shown to lead to steric clashes with α helix 2 (α2) of the PXR LBD, resulting in decreased binding affinity and, consequently, lower CYP3A4 induction.^20^ To develop rifamycin-based antibacterials with reduced CYP induction, rifabutin was selected as the starting scaffold because clinical studies show substantially lower CYP3A4 induction,^21,22^ and more importantly, lower DDIs^23–26^ with rifabutin than rifampicin. Notably, however, rifabutin can exhibit comparable or even greater induction than rifampicin in primary hepatocyte assays, consistent with differences between intrinsic PXR activation measured in vitro and exposure-dependent effects observed in vivo.^27^ A set of rifabutin analogs with a diversity of substituents and linkers at the C25 position (i.e., ester and carbamate) were designed and synthesized (Table 1). The synthesis of esters **1**–**3** and carbamates **6**, **9**, and **10** were reported previously.^19^ The published carbamate route was also utilized to prepare **4**, **5**, **7**, **8**, and **11**.^28,29^ The ability of all analogs to inhibit the growth of in vitro cultured *Mycobacterium abscessus* ATCC 19977 was previously reported^15,19^ to be characterized by minimum inhibitory concentration (MIC) values in the range of 15–67 nM (Table S1). The ability of these analogs (at a test concentration of 10 μM) to induce cellular CYP3A4 expression in in vitro cultured human hepatocytes was evaluated by reverse transcription quantitative real-time polymerase chain reaction (RT-qPCR).^30^ Rifampicin and flumazenil, a clinical CYP noninducer, were tested as positive and negative controls, respectively, at 10 and 30 μM. Cryopreserved hepatocytes were obtained from different donors and assayed in separate batches. To enable cross-batch comparison, CYP3A4 induction values were normalized within each experiment using vehicle control as the baseline (0%) and rifampicin as the positive control (100%). All reported induction values, therefore, represent relative PXR activation compared to rifampicin. For example, the value of CYP3A4 induction of flumazenil, the negative control, was 3.3% of rifampicin (Table 1). As anticipated, the normalized value of CYP3A4 induction of 131.3 for rifabutin was considerable. Switching the rifabutin C25 acetyl group with benzoyl or heteroaroyl groups, as shown in compounds **1**–**3**, led to significant decreases in normalized CYP3A4 induction level as compared to the parent drug rifabutin. A more pronounced reduction in CYP3A4 induction was observed with carbamate analogs **4–11**. The carbamate N-substituent featured a range of cores such as piperidine (cf., **4**, **5**), a fused ring system (cf., **6**, **7**), nitrogen-containing aromatic heterocycles (cf., **8**, **9**), or a thiazole (cf., **10**, **11**). Taken together, these results demonstrate that bulky C25 modifications can significantly reduce CYP3A4 induction by the rifabutin scaffold.

**Table 1:**
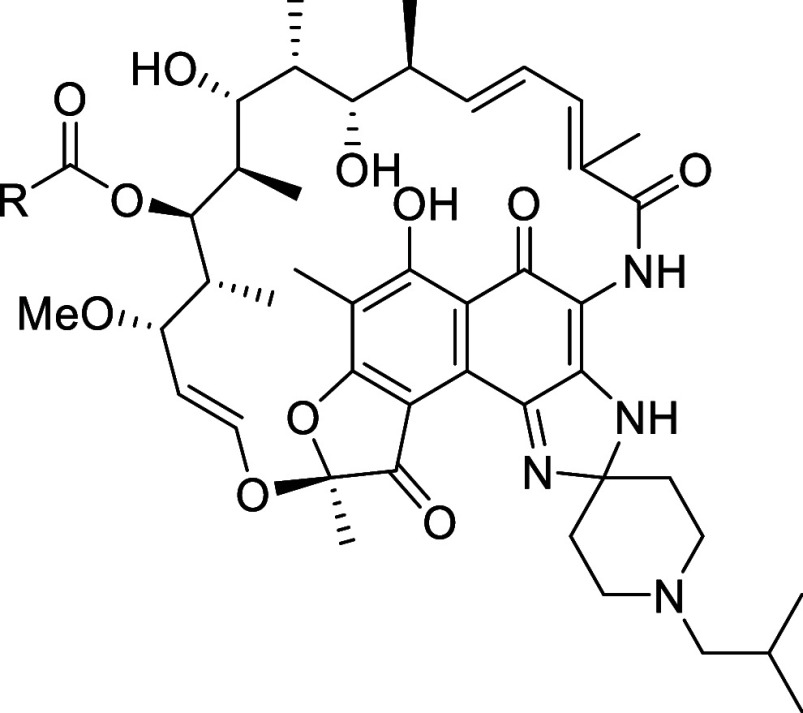

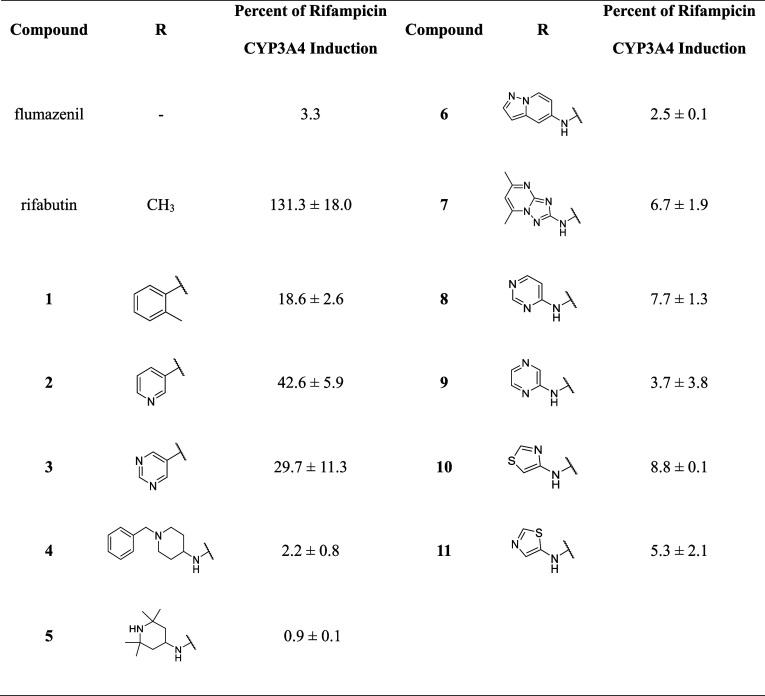
Normalized CYP3A4 Induction Levels of C25 Modified Analogs in Primary Human Hepatocytes^a^

a The data are normalized to DMSO (0%) and 10 μM rifampicin (100%).

### Molecular Docking Studies Suggest that Bulky C-25 Substitutions Perturb the Binding Pose and Disrupt Key Interactions within the PXR LBD

To explore whether attenuated CYP3A4 induction observed with these C25-modified rifabutin analogs is associated with disrupted PXR binding, we conducted molecular docking studies using the PXR LBD structure from the previous rifampicin-PXR LBD X-ray cocrystal structure (PDB ID: 1SKX). This structure lacks electron density for the 4-methyl-1-piperazinyl ring of rifampicin and is missing protein residues 178–209, 229–235, and 310–317. To create a complete model, these missing residues were reconstructed using AlphaFold2.^31^ The resulting model was superimposed onto the 1SKX crystal structure to confirm agreement with the experimental structure (Figure S1). Rifabutin was docked and overlaid with the original rifampicin pose from PDB ID: 1SKX. Rifabutin’s lowest binding energy pose overlapped with that of rifampicin (root-mean-square deviation (RMSD) of 0.27 Å), consistent with its PXR agonist effect (Figure 2A). Next, we docked compound **4**, a C25-modified rifabutin analog featuring a 4-benzylpiperidyl carbamoyl group, into the PXR LBD structure as a representative noninducer. The lowest binding energy pose of compound **4** exhibited a significantly different orientation, with a large RMSD of 11.9 Å with respect to rifabutin (Figure 2B).

**Figure 2:**
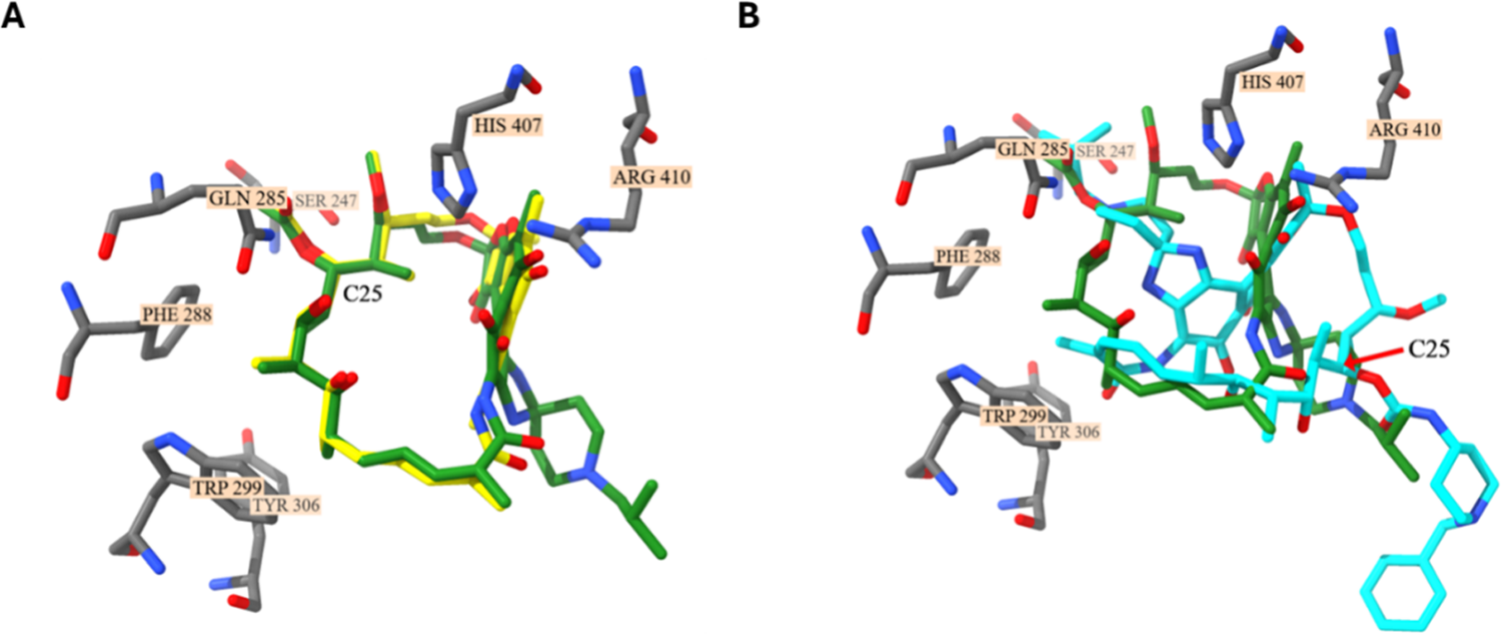
(A) Overlap of binding poses of rifampicin (yellow, PDB ID: 1SKX) and docked rifabutin (green). (B) Overlapped molecular docking results of rifabutin (green) and **4** (cyan) in PXR LBD. This figure was created with UCSF ChimeraX (Version 1.11).

Spurred by these initial results, we docked rifabutin and analogs **1**–**11** into the full PXR LBD structure. To evaluate the docking results, we analyzed the docking scores for the lowest energy conformer of each analog, alongside predicted hydrogen-bonding interactions with key residues Gln285, His407, and Arg410, and predicted hydrophobic interactions with Phe288 and Trp299 (Figure 3). These residues were chosen because they participate in the interaction network observed for rifampicin in the 1SKX X-ray crystal structure and have been identified as significant ligand-recognition hotspots in the PXR LBD.^32^ Trp299 and Phe288 form part of a hydrophobic “cage”, with Gln285 at the adjacent polar rim, that anchors many cocrystallized ligands. His407 and Arg410 belong to a complementary hotspot on the opposite side of the pocket that can form key hydrogen bonds with bound ligands.

**Figure 3:**
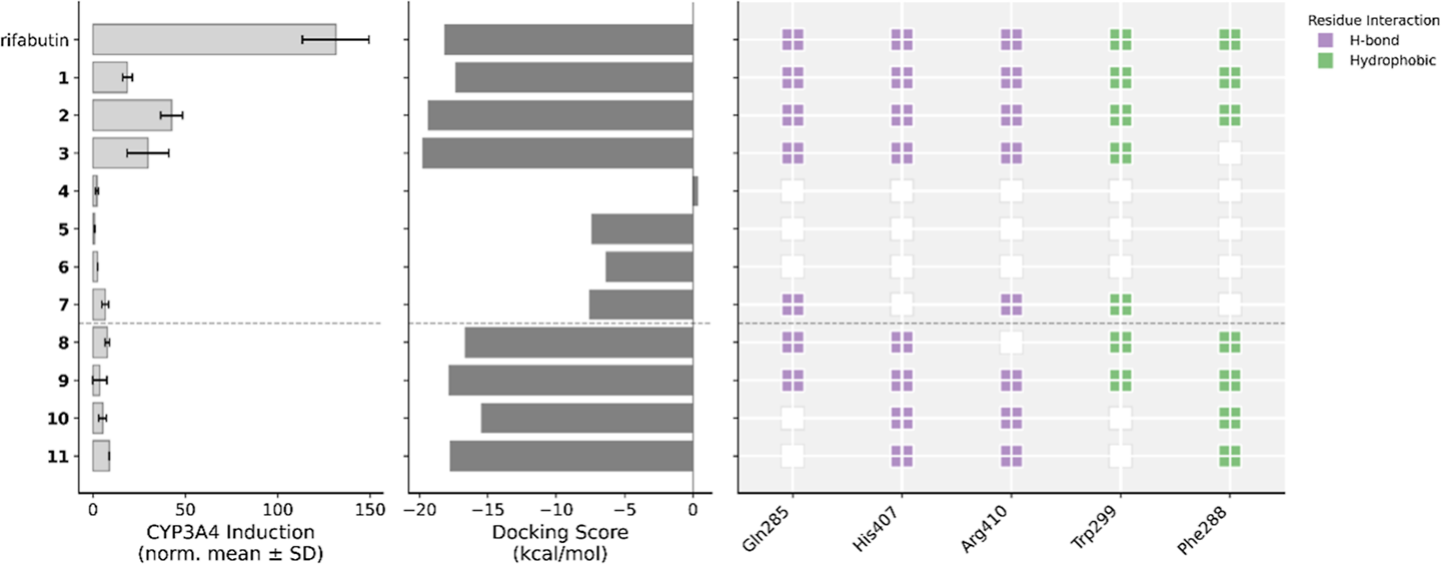
Comparison of experimental CYP3A4 induction and molecular docking results for rifabutin and analogs **1–11.** The left panel displays normalized CYP3A4 induction levels shown as mean ± SD from Table 1. The middle panel shows the predicted docking scores (kcal/mol) for the lowest energy conformer of each compound. The right panel provides an interaction map of predicted molecular interactions within the PXR LBD, where purple squares indicate proposed hydrogen-bonding (H-bond) interactions and green squares indicate proposed hydrophobic interactions. Key residues evaluated include Gln285, His407, and Arg410 for H-bonding, and Trp299 and Phe288 for hydrophobic contacts. The horizontal dashed line separates the final four compounds (**8**–**11**) where the docking scores were not consistent with the experimental CYP3A4 induction data. This figure was created with Python (Version 3.9).

Docking studies revealed that rifabutin and compounds **1**–**3** docked in the PXR LBD in a manner consistent with their higher CYP3A4 induction values. These compounds yielded docking scores ranging from −19.4 to −17.4 kcal/mol and they were predicted to maintain key interactions within the PXR LBD. However, compound **3** was not predicted to interact with Phe288. In contrast, compounds **4**–**7**, representing a subset of the weaker CYP inducers, were predicted to bind with significantly higher (i.e., poorer) docking scores ranging from −7.4 to 0.38 kcal/mol. Compounds **4**–**6** were predicted to lose all assessed key interactions with the PXR LBD, while compound **7** lacked interactions with His407 and Phe288. Notably, the lowest energy poses for compounds **4**, **5**, and **7** were rotated approximately 180° relative to the orientation of rifabutin and rifampicin, with the C25 group facing helix 2 and the isopropyl tail oriented toward residues Gln285 and Phe288 (Figure S2). This suggests that bulky substitutions at C25 may cause steric clashes with the protein during docking, forcing the molecule into these alternate relatively low-energy conformations. Compounds **10** and **11** displayed docking scores similar to rifabutin but were not predicted to form the key H-bond with Gln285 or the hydrophobic interaction with Trp299. The docking results for compounds **8** and **9** were the least consistent with the experimental induction data as they maintained docking scores and key protein interactions similar to those of rifabutin despite their low induction profiles.

### PXR Binding Affinity Was Not Abolished by Rifabutin C25-Modification

To experimentally investigate the effect of rifabutin C25-modification on PXR engagement, we measured the binding of analogs to the PXR LBD using a time-resolved fluorescence resonance energy transfer (TR-FRET) assay,^33^ in which the test compound competitively binds to the PXR LBD and displaces a FRET-inducing PXR ligand leading to FRET signal inhibition (Table 2). T0901317 was utilized as a positive control.^34^ Rifabutin and rifampicin exhibited maximum binding (*I*_max_) of 64% and 66%, respectively, at the highest assay concentration of 30 μM. The IC_50_ values of rifabutin and rifampicin, indicative of their binding affinities, were comparable to previously reported values.^20^ The PXR binding of C25-modified rifabutin analogs afforded two clusters as grouped by binding affinity (Figure 4). Group A (**2**–**6**, **8**, **10**) exhibited substantially lower *I*_max_ values, indicating decreased PXR binding (IC_50_ > 30 μM) as compared to the remainder of the analogs which were placed in Group B (**1**, **7**, **9**, **11**) with *I*_max_ levels comparable to rifabutin. Within Group B, **7**, **9** and **11** had similar binding affinities to rifabutin, while **1** exhibited nearly a 1 log_10_ higher binding affinity than rifabutin.

**Table 2:**
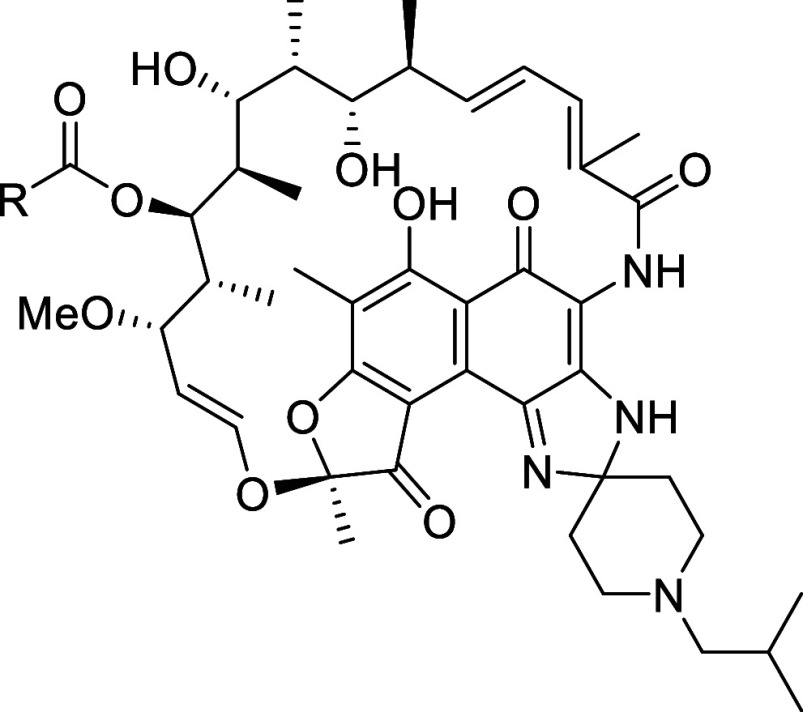

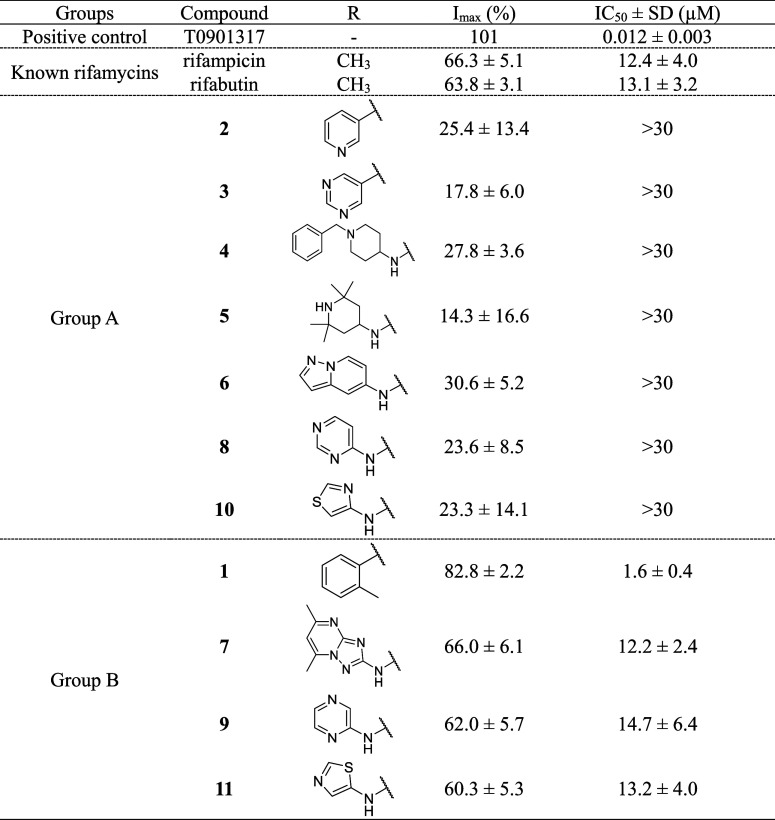
In Vitro PXR LBD Binding Affinities of Rifamycins and C25-Modified Rifabutin Analogs

**Figure 4:**
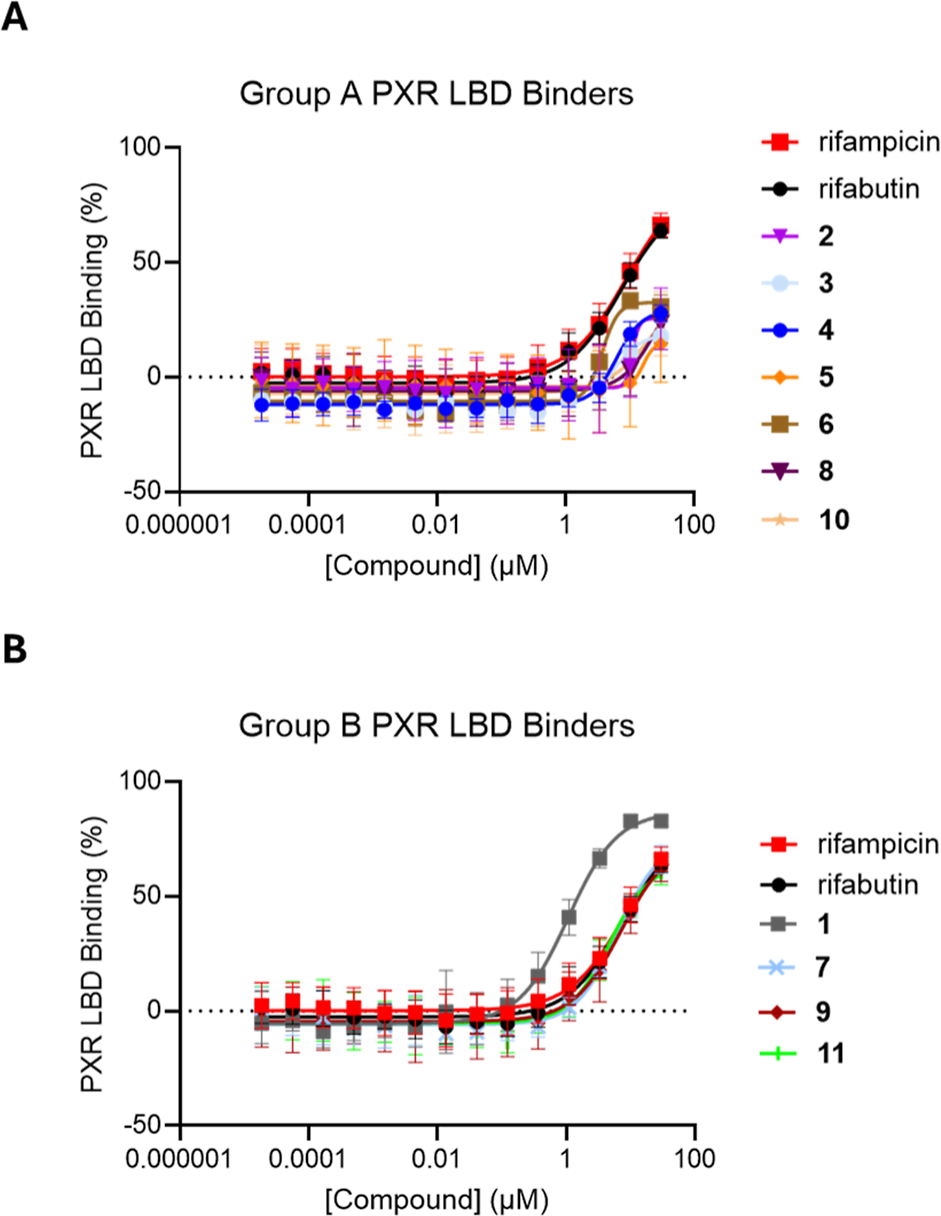
C25-Modified rifabutin analogs cluster into groups (A) and (B) based on their PXR binding properties. T0901317 (10 μM) was utilized as the 100% normalization control.

### Modification of Ansa-Chain Changes PXR Responses

The decreased PXR binding affinity of the analogs in Group A may account for their reduced CYP3A4 induction in the human hepatocyte assay (Table 1). However, this correlation was not established for Group B analogs. Compounds **7**, **9** and **11** had similar PXR binding affinities as compared to rifabutin, and yet these three analogs exhibited significantly reduced CYP3A4 induction. This unique behavior of **7**, **9** and **11** prompted us to further explore the mechanism of their abrogation of CYP3A4 induction. Ligand–receptor binding can lead to different target responses including agonism (i.e., elicit target response similar to endogenous mediators which is usually stimulation), inverse agonism (i.e., elicit target response opposite to agonists), or antagonism (i.e., bind to the target without initiating further responses). Changing the target response of a ligand can be achieved by minor structural changes, such as adding/removing a chemical group^35^ or repositioning a chemical group.^36^ Indeed, this has been shown for PXR itself, with minor changes in certain scaffolds leading to agonism, inverse agonism, and antagonism.^37–41^ Therefore, we hypothesized that for **7**, **9** and **11**, the modifications at C25 convert their pharmacology from a PXR agonist to an antagonist or inverse agonist. To test this hypothesis, we assayed these three analogs in a PXR-responsive cellular reporter assay using HepG2 cells that were transiently transfected with a PXR-expressing plasmid and a PXR-responsive CYP3A4 promoter-controlled luciferase reporter.^42^ Using this cellular PXR activation assay, rifampicin and rifabutin behaved as agonists with EC_50_ values of ∼0.3 μM (Figure 5A). For the three C25-modified rifabutin analogs, **9** appeared essentially inactive while **7** and **11** displayed inverse agonist-like behavior (Figure 5A). Notably, all three analogs reduced PXR activation by rifabutin (Figure 5B). Taken together, these results suggest that the C25 modifications in analogs **7**, **9** and **11**, instead of abrogating PXR binding, may convert rifabutin from an agonist to an antagonist in the case of **9** or inverse agonist (cf., **7** and **11**), thereby significantly attenuating CYP3A4 induction.

**Figure 5:**
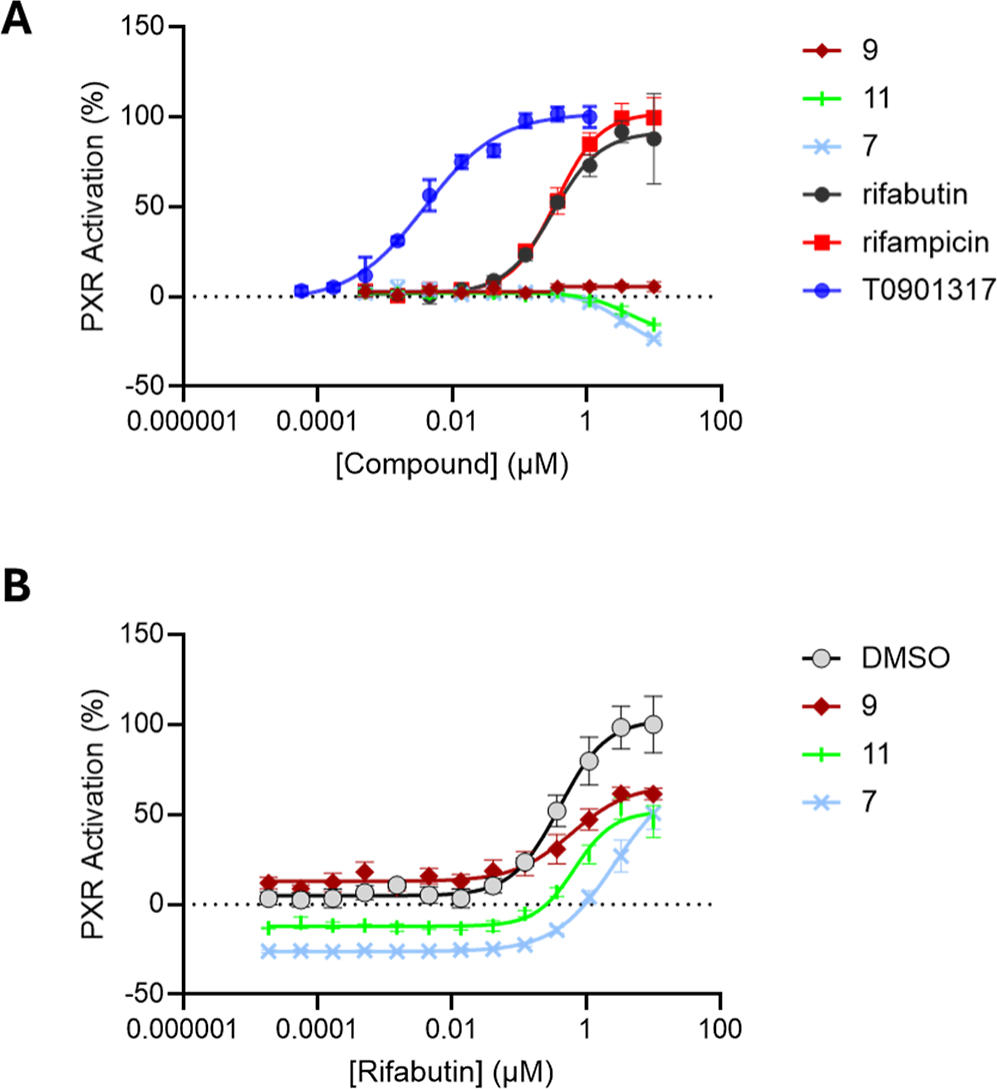
Cellular PXR-responsive reporter assay identifies rifabutin analogs with converted PXR response. (A) PXR reporter assays were performed in dose response for the indicated compounds. Compared to known PXR agonists rifabutin, rifampicin, and T0901317, **9** showed activity loss while **7** and **11** showed behavior consistent with inverse agonists. (B) PXR reporter assays were performed in dose response for rifabutin alone or in the presence of 10 μM **9**, **11** or **7**. The three analogs reduced PXR activation by rifabutin, with **11** and **7** also showing inverse agonist character (PXR activation values below 0%). DMSO was utilized as the negative control (0% PXR activation; basal activity) and T0901317 served as the positive control (1 μM T0901317 afforded 100% PXR activation; full activation).

### Molecular Dynamics Simulations Suggest Asymmetric α12 Displacement as a Metric for Differentiating Rifabutin Analog Agonists and Antagonists of PXR

Given the divergent responses observed for compounds **7**, **9**, and **11** in PXR activation assays despite comparable apparent binding affinities, we further probed these ligand-dependent differences in binding behavior with molecular dynamics (MD) simulations. Three compounds were selected for the simulations: rifabutin (agonist), **9** (potential antagonist) and **7** (potential inverse agonist). Simulations were performed in GROMACS (version: 2025.4) for a duration of 200 ns in duplicate for each ligand using the published X-ray crystal structure of the PXR LBD (PDB ID: 1SKX) with modeled missing loops and rifampicin removed. Analysis of the simulations consisted of two major phases. The first phase involved characterizing ligand-dependent conformational states of the PXR helices. A particular focus was placed on α helix 12 (α12), as it plays an established role in modulating PXR activity by mediating interactions with LXLL motifs on steroid receptor coactivator-1 (SRC-1),^43^ thereby influencing transcriptional regulation of PXR target genes.^44^ Recent studies have further shown that ligand-dependent perturbations of α12 can be captured in MD simulations comparing agonists and antagonists.^40,45^ The second phase of the analysis focused on ligand–protein interactions at the residue level, including residues such as Ser247 and Trp299, which have previously been shown to significantly reduce PXR-mediated CYP activation by rifamycins and other PXR ligands when mutated.^12,20^

The first and last frames from each simulation were overlaid with 1SKX to investigate changes in the position of α12 induced by each ligand (Figure 6). For rifabutin, α12 in the final frame adopted a modestly outward-shifted conformation relative to both the initial frame and the 1SKX X-ray crystal structure. In contrast, compound **9** induced an asymmetric displacement characterized by outward movement of the N-terminal region of α12, while the C-terminus remained largely unchanged. Compound **7** produced a more pronounced asymmetric perturbation, with substantially greater displacement of the N-terminal region compared to the C-terminus. To quantify this asymmetry, α12 was divided into two regions: an N-terminal segment (residues 423–426) and a C-terminal segment (residues 427–430). For each region, the average Cα–Cα distance to six anchor residues (Leu281, Glu282, Val284, Gln285, Lys286, and Phe288) was calculated (Figure 7). Anchor residues were selected from the lowest 10% of root mean-square fluctuation (RMSF) values for amino acid residues over the course of all six MD simulations to minimize contributions from anchor flexibility (Figure S3). For each trajectory, the difference between the mean distances of the C- and N-terminal α12 segments was calculated as the α12 asymmetry metric Δ = *d*_C_ – *d*_N_, providing a quantitative measure of asymmetric α12 motion.

**Figure 6:**
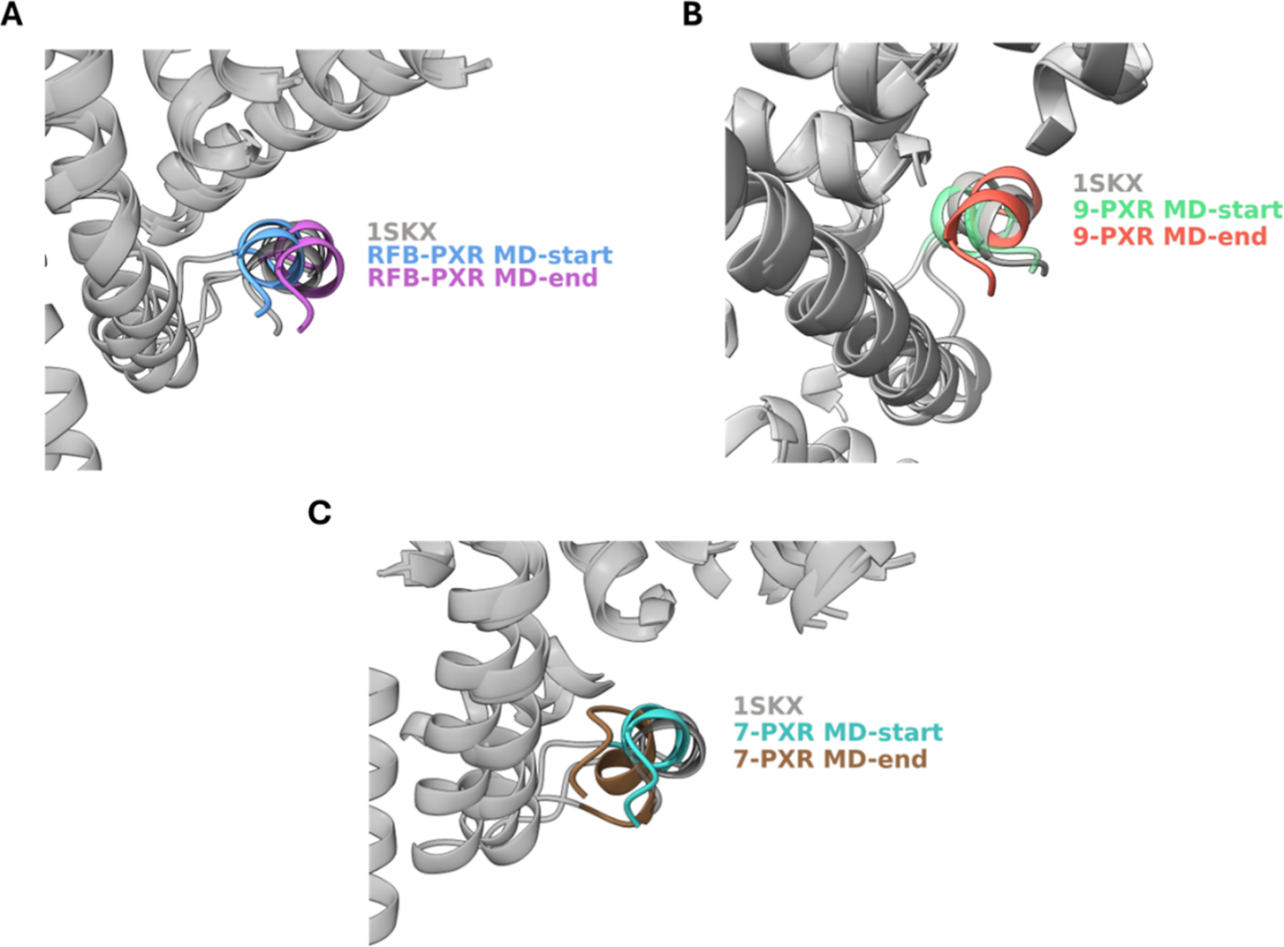
Ligand-dependent perturbation of PXR α12 observed in MD simulations. Structural overlays of the α12 helix from the PXR LBD are shown for rifabutin (RFB) (A), compound **9** (B), and compound **7** (C). For each ligand, the published PXR LBD-rifampicin crystal structure (PDB ID: 1SKX, gray) was superimposed onto the initial (MD-start) and final (MD-end) frames from 200 ns MD simulations using Cα backbone alignment. This figure was created with UCSF ChimeraX (Version 1.11).

**Figure 7:**
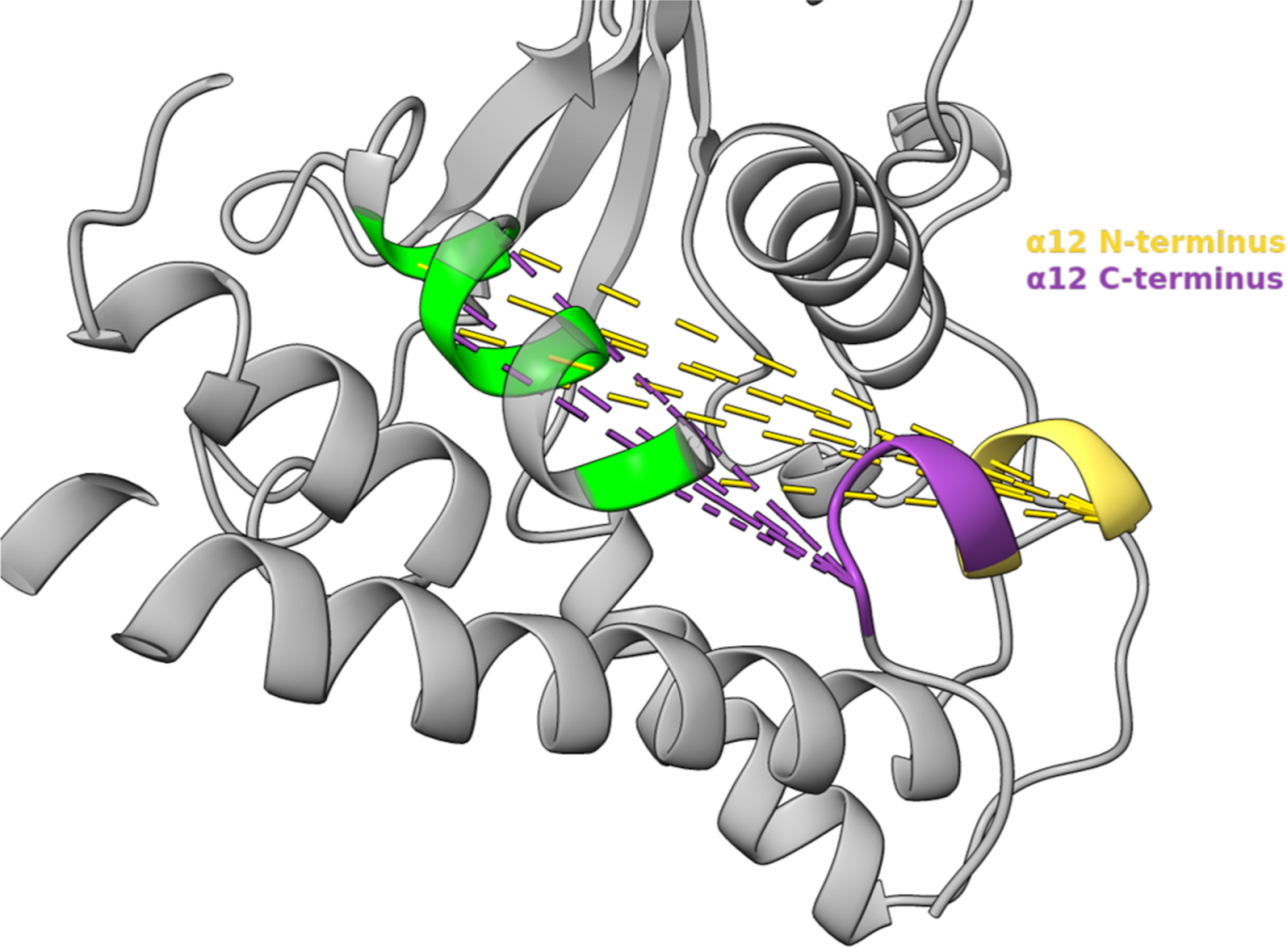
Quantification of α12 asymmetry metric. Schematic representation of the α12 asymmetry metric divided into N-terminal (yellow) and C-terminal (purple) segments, illustrating the calculation of average Cα–Cα distances from each segment to a set of low-RMSF anchor residues (Leu281, Glu282, Val284, Gln285, Lys286, and Phe288) colored green. Distances are shown for one representative residue from each segment of the α12 helix, although the metric is calculated as the average across all residues in the respective N- and C-terminal regions. This figure was created with UCSF ChimeraX (Version 1.11).

α12 Exhibited the greatest average asymmetric displacement in the presence of the potential inverse agonist **7** across both replicates, followed by the potential antagonist **9**, with rifabutin showing the smallest average asymmetry (Table 3). Time-resolved analysis (Figure S4) further revealed distinct dynamic behaviors among the ligands: rifabutin displayed substantial fluctuations in α12 displacement, with values spanning a wider range (between approximately 0 and −4), indicative of increased conformational flexibility. In contrast, both **7** and **9** maintained persistently low negative values, consistent with stabilization of an asymmetric α12 conformation.

**Table 3:** Statistics for α12 Asymmetry Metric Across Ligands and Replicates^a^

	mean (Å)	SD (Å)
RFB—replicate 1	–1.7	1.2
RFB—replicate 2	–1.5	0.99
**9**—replicate 1	–3.9	0.46
**9**—replicate 2	–3.3	0.70
**7**—replicate 1	–4.4	0.97
**7**—replicate 2	–4.7	0.94

a Mean and standard deviation (SD) of the α12 asymmetry metric (Δ = *d*_C_ – *d*_N_) calculated over 200 ns MD simulations for rifabutin (RFB), compound **9**, and compound **7**.

To further contextualize these simulation-derived values, the α12 asymmetry metric was calculated for a set of published crystal structures of PXR LBD with different ligands bound (Table S2). Since all PXR LBD structures have the canonical “active” α12 conformation, calculated asymmetry metric values were similar across examined X-ray crystal structures (∼average of −0.85) regardless of the ligand bound. The metric suggests that α12 positioning in the X-ray crystallography-defined states is generally closer to a symmetric configuration similar to that observed in MD simulations with rifabutin, in contrast to the potential antagonist **9** and inverse agonist **7**, which resulted in more pronounced asymmetry during MD simulations.

Next, the first and last frames of each simulation were overlaid with the 1SKX structure to determine whether α12 helix motion could be explained by steric interactions arising from bulky C25 substitutions. Simulation end frames revealed that bulky substituents at C25, especially in the case of **7**, are predicted to directly clash with residue Phe251 and perturb the general orientation of Phe429 (Figure 8), the latter located on α12. Phe251 was immediately adjacent to α12 and has previously been used as a reference residue for monitoring perturbations in α12 positioning.^40^ These interactions offer a structural rationale for the outward displacement and increased asymmetry of α12 observed in the simulations.

**Figure 8:**
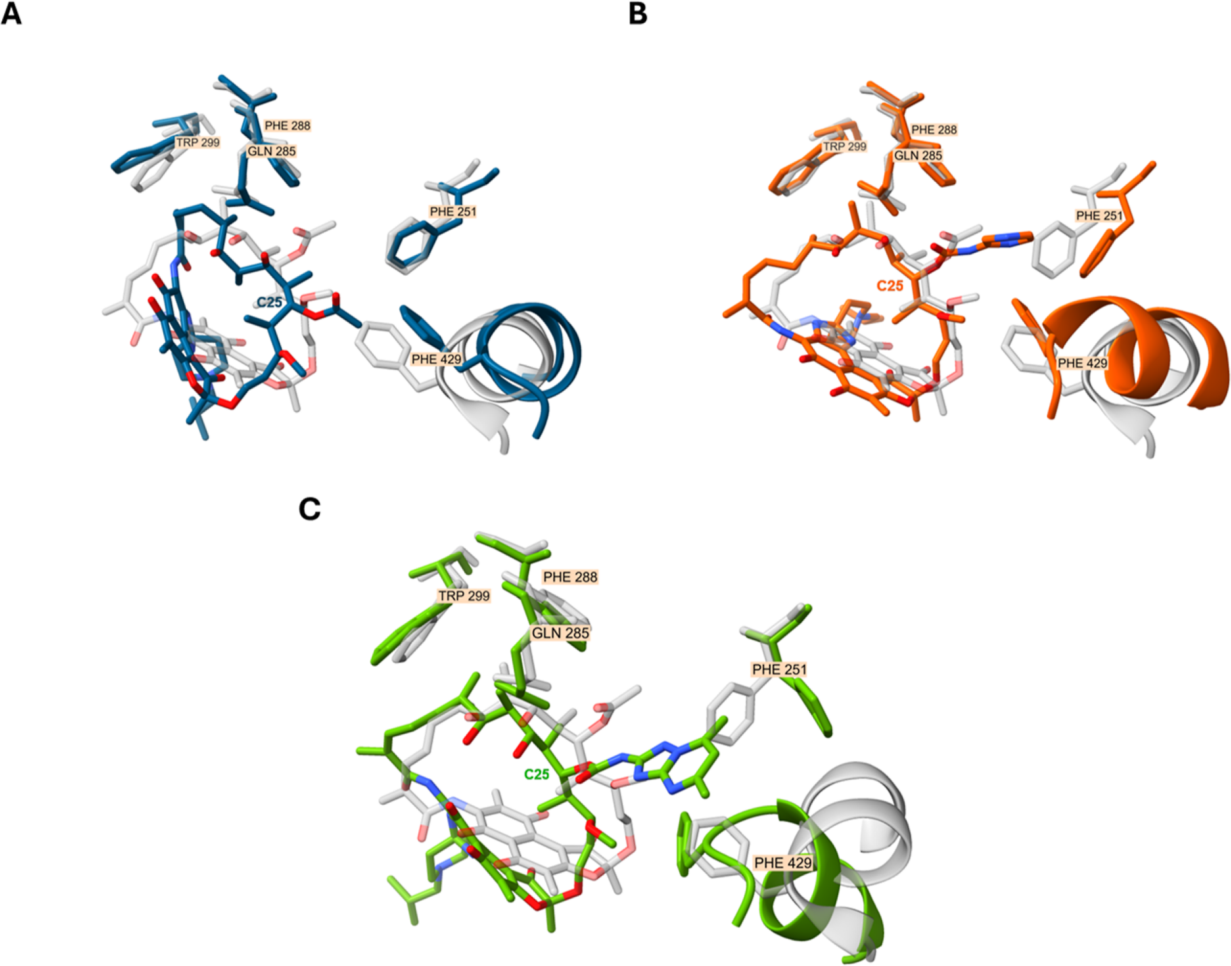
Overlay of the final simulation frame for each ligand with the 1SKX X-ray crystal structure. Structural overlays of rifabutin (blue), **9** (orange), and **7** (green) last frames from MD simulations with 1SKX (gray). α12 is shown for reference. Compounds **9** and **7** lead to significant movement in Phe251, in likelihood due to unfavorable steric interactions with the compounds. Perturbations in the orientation of Phe429 are also observed. This figure was created with UCSF ChimeraX (Version 1.11).

Next, we examined hydrogen-bonding and hydrophobic interactions between ligands and PXR LBD over the course of the simulations. Across all systems, ligands formed a comparable number of hydrogen bonds and hydrophobic contacts (Figures S5 and S6) with the receptor, with no single ligand exhibiting a consistently higher or lower interaction count relative to the others. The observed fluctuations reflect the dynamic nature of the PXR ligand-binding pocket and its ability to accommodate a diversity of ligands. Analysis of hydrogen bonds revealed that all three ligands engage a common set of residues, including Arg410, Gln285, His407, and Ser247, albeit with varying occupancies throughout the simulations. These residues have previously been implicated in rifamycin binding to PXR^46^ and established as common hydrogen-bonding residues for PXR ligands.^32^ Among rifabutin, **9**, and **7**, the major difference between their predicted H-bonding network with the PXR LBD was an interaction with Ser208 observed almost exclusively for rifabutin (fractional occupancy 0.46 for RFB, 0 for **9**, and 0.01 for **7**). As Ser208 is a regulatory phosphorylation site implicated in modulating cofactor interactions required for gene expression,^47^ this interaction may contribute to the stronger transcriptional activation observed for rifabutin. Differences also emerged in the pattern of hydrophobic contacts between ligands (Supporting Information Figure S7). Both **9** and **7** exhibited more persistent hydrophobic interactions involving their C25 modifications, particularly with residues Phe251 and Phe429. These observations, combined with the structural overlays, highlight that although these contacts are dynamic, their increased persistence in the antagonist and inverse agonist simulations may contribute to the altered α12 positioning observed in these simulations. Altogether, these simulations support the hypothesis that the different transcriptional responses observed may arise from α12 asymmetry or, more broadly, perturbations in α12 positioning. Importantly, the observations suggest a framework to inform further optimization of this compound series.

## Discussion

DDIs contribute substantially to adverse health outcomes and preventable healthcare costs, many of which arise from modulation of CYP enzymes.^48,49^ CYP3A4 is responsible for the metabolism of a large proportion of clinically used drugs and significant DDIs have been documented when its expression is altered by inducers and inhibitors.^50^ PXR is one of the primary regulators of CYP3A4 expression, and understanding the molecular mechanisms by which ligand–receptor interactions give rise to distinct transcriptional responses is critical. Compared to other nuclear receptors, PXR LBD has a large binding pocket with a volume of 1200–1600 Å^3^,^51^ which allows PXR to accommodate ligands, and thus xenobiotics, with a wide range of sizes and chemical functionality. However, PXR ligand binding is not completely promiscuous as certain structural elements within PXR have been found to directly control ligand binding affinity. For example, two PXR regions, the α2–β1 loop and the Phe288–Trp299–Tyr306 π-trap, have been found to significantly contribute to ligand binding, and introducing steric clash with either of these two regions by chemical modifications of a known PXR activator resulted in loss of binding affinity.^20^ On the other hand, PXR-mediated gene regulation relies on a highly complicated and dynamic network of protein–protein and protein-nucleic acid interactions. Full execution of PXR function requires formation of the PXR-RXR heterodimer^43^ and recruitment of coactivator proteins.^11^ This complex mechanism of PXR function indicates that ligand binding itself is not sufficient and certain conformational changes of PXR must be achieved to trigger subsequent interactions with coregulators. Ligands that bind to PXR but fail to initiate this required conformational change will likely lose the function of PXR agonism and act instead as PXR antagonists. Conversely, inverse agonists induce conformational changes dissimilar to those of agonists, that promote corepressor recruitment and gene repression. In line with this, regulatory structural elements within PXR have been connected to PXR response to ligand binding. Among these regulatory elements is α12 of PXR, which has been shown to occupy a central structural role in determining ligand-dependent transcriptional outcomes, including agonism and antagonism through interactions with coactivator SRC-1.^45^ Furthermore, PXR ligands inducing different conformations of α12 were previously shown to have opposite PXR responses (i.e., agonism versus antagonism).^40,45^ Taken together, the presence of the ligand binding- and PXR response-determining regions suggests that rational modifications targeting these regions offer a promising strategy to eliminate PXR-mediated DDIs of drug molecules via either attenuating PXR binding affinity or altering the downstream PXR response.

Structure-based optimization to remove PXR activation has been successfully implemented in medicinal chemistry campaigns for various therapeutic targets.^32^ However, almost all of these examples concentrated on novel small molecule leads, whereas few reports have focused on optimization of currently approved drugs to eliminate their undesired PXR-mediated DDIs. As one of the most important antimycobacterial drugs, rifampicin is also a potent PXR agonist leading to significant DDIs with a diverse panel of drugs. Rifampicin was among the first reported PXR agonists nearly 30 years ago^9^ and yet a CYP induction-free rifamycin drug remains nonexistent. Our work herein presents a structure-based and MD-guided strategy of eliminating CYP induction from rifabutin. Through modifications on the ansa-chain moiety, we altered the original binding modes of rifabutin in PXR, which resulted in a marked decrease of PXR binding affinity or a potential switch of the ligand pharmacology from agonism to antagonism or inverse agonism.

Docking analysis suggests a structural basis for this behavior. C25 modifications are predicted to perturb the canonical rifampicin binding pose (PDB ID: 1SKX), likely driven by steric clashes and the loss of key protein interactions, which may contribute to reduced CYP3A4 induction. However, docking failed to reconcile the experimental results observed for compounds **7** and **9**, highlighting the inherent complexity of PXR-ligand binding. To investigate this discrepancy, we performed MD simulations for rifabutin and both analogs. The simulations predict that ligand-dependent changes in α12 positioning may provide a possible explanation for reduced CYP3A4 induction despite binding affinity comparable to rifabutin (Table 2).

## Conclusions

Although the conversion of receptor response by subtle changes of ligand structure has been observed for many types of receptors including PXR,^40^ our work presents an important example of this ligand pharmacology conversion for the prototypical PXR ligands of the rifamycin family. This knowledge will benefit the further pursuit of rifamycin drugs, as well as other drug classes, devoid of significant CYP3A4 induction that is implicated in problematic DDIs.

## Experimental Section

### Chemistry

All synthesized molecules for biological assessment were of >95% purity as demonstrated by LC trace.

### Computational Chemistry Methods

#### Molecular Docking Analysis

The computational modeling approach was adapted from Ashkar et al.^13^ The X-ray structure of rifampicin bound to PXR LBD was obtained from the Protein Data Bank (PDB ID: 1SKX). This structure lacks electron density for the 4-methyl-1-piperazinyl ring of rifampicin and is missing protein residues 178–209, 229–235, and 310–317. The missing residues 178–209, 229–235 and 310–317 were modeled using AlphaFold2 ^31^ with existing PXR structures as a guide. The protein structure was then prepared for docking simulations using the QuickPrep function in the Molecular Operating Environment (MOE) software package (Version 2024.06). QuickPrep was used to correct topological errors in several residues (e.g., incorrect hybridization). Hydrogen atoms were added using Protonate3D. Tethers were installed on binding pocket, ligand, and solvent atoms present in the binding pocket to limit their deviation from the experimentally determined coordinates during simulations. Atoms further away from the binding pocket were fixed to increase efficiency during docking calculations. Refinement and energy minimization of the system were performed using the Amber:EHT force field. The missing methyl piperazine tail of rifampicin in the 1SKX structure was added using the Builder function in MOE. The resulting rifabutin structure was minimized using the Merck molecular force field 94× (MMFF94×). The same approach was used for all rifabutin analogs that were docked. Post minimization, LowModeMD was used to generate conformers for each compound. The resulting conformers were docked into the PXR LBD structure using the built-in Triangle Matcher placement function in MOE, employing London Δ*G* for initial placement and GBVI/WSA Δ*G* for refinement. To evaluate the docking results, we analyzed the docking scores for the lowest energy conformer of each analog, alongside predicted hydrogen-bonding interactions with key residues Gln285, His407, and Arg410, and predicted hydrophobic interactions with Phe288 and Trp299.

#### MD Simulations

For each ligand, the following approach was taken. The initial ligand pose was obtained from the lowest energy docked conformation with MOE. The molecule and its three-dimensional coordinates were then parametrized using the antechamber suite in AmberTools23, applying the AM1-BCC charge model with the general AMBER force field (GAFF2).^52^ GROMACS-compatible ligand topology files were generated using ACPYPE.^53^ Prior to protein topology generation, docking-related restraints were removed, and the protein was energy-minimized in MOE using the Amber:EHT force field to ensure consistent starting points across ligands. All simulations were performed in GROMACS (Version 2025.4).^54^ Protein–ligand complexes were placed in truncated dodecahedral simulation boxes with a minimum solute-box distance of 10 Å and solvated using the CHARMM-modified TIP3P water model. System charge was neutralized by addition of counterions. Energy minimization was carried out using the steepest descent algorithm for up to 50,000 steps or until the maximum force fell below 1000 kJ·mol^–1^·nm^–1^. Electrostatic interactions were treated using the particle mesh Ewald (PME) method. Neighbor searching employed the Verlet cutoff scheme with a cutoff of 1.2 nm and a neighbor list update frequency of 20 steps. All bonds involving hydrogen atoms were constrained using the LINCS algorithm, and a time step of 2 fs was used throughout. Following minimization, systems were equilibrated under *NVT* conditions at 300 K using the velocity-rescale (V-rescale) thermostat. This was followed by *NPT* equilibration at 300 K and 1 bar using the Parrinello–Rahman barostat. Nonbonded interaction parameters were retained during equilibration with both electrostatic and van der Waals cutoffs set to 1.2 nm. Equilibration was assessed by monitoring temperature, density, box dimensions, volume, and potential energy (Table S3). Production MD simulations were initiated following equilibration and conducted at 300 K and 1 bar using the same thermostat, barostat, and interaction parameters using the CHARMM general force field (CGenFF).^55^ Periodic boundary conditions were applied in all three dimensions. To assess system stability, time-resolved profiles for protein backbone RMSD (Å), protein radius of gyration, intraprotein H-bonds and ligand RMSD (Å) were calculated (Figure S7). Analyses were performed in Python 3.9. Data manipulation was carried out with NumPy and pandas, and visualizations were generated using Matplotlib and seaborn. The MD analysis code may be accessed at https://github.com/freundjs/Public-Freundich-Lab/tree/main/rifamycin-structural-modifications-attenuate-drug-drug-interactions.

#### Selection of Anchor Residues for the α12 Asymmetry Metric

Cα RMSF were computed from the GROMACS simulation files. Terminal residues were excluded from consideration to avoid chain-end flexibility confounding distance metrics. Residues in the bottom 10% of RMSF values in all 6 simulations were identified and residues Leu281, Glu282, Val284, Gln285, Lys286, and Phe288 were selected since they were all located on the same helix (Figure S3).

#### α12 Asymmetry Metric Calculation

Distances were calculated between α12 residues 423–430 and selected rigid anchors. Residues 431–434 were excluded as they were part of the highly flexible C-terminal region of α12 (Figure S8). The helix was subdivided into N-terminal (423–426) and C-terminal (427–430) halves and the hinge asymmetry metric at each frame was defined as

$$\Delta =\left(d_{C}\right)-\left(d_{N}\right)$$

where each term represents the mean distance (Å) from the corresponding α12 half to the rigid anchor set.

#### Hydrogen-Bond and Hydrophobic Contact Analysis

Protein–ligand hydrogen bonds and hydrophobic contacts were analyzed over the full production MD trajectories in Python using MDAnalysis.^56^ Hydrogen bonds between ligands and PXR were identified using standard geometric criteria of maximum donor–acceptor distance of 3.5 Å and hydrogen-donor–acceptor angle cutoff of 30°.^57^ Hydrophobic contacts were quantified using a distance-based approach focusing exclusively on heavy atoms to avoid bias from hydrogen placement. A hydrophobic contact was defined as any instance in which a ligand heavy atom was within 4.0 Å of a nonpolar protein side-chain heavy atom. Contact and H-bond occupancies were computed as the fraction of trajectory frames satisfying each criterion for protein–ligand pairs.

### Biological Methods

#### Primary Human Hepatocyte Assays

Cryopreserved human hepatocytes (male or female, one donor) were thawed and plated into collagen-coated 96-well plates in InVitroGR CP plating medium (BioIVT, cat. #Z99029) at a density of 0.7 × 10^5^ viable cells/mL. The hepatocytes were cultured at 37 °C and 5% CO_2_ for 18–24 h. The hepatocytes were washed with PBS and incubated with the test compound in serum-free InVitroGRO HI maintenance medium (BioIVT, cat. #Z99009), which was changed daily for a total incubation of 72 h. On day 5 after plating, the hepatocytes were washed and lysed by RT-qPCR Sample Preparation Reagent (Bio-Rad, cat. #170-8899). The cell lysate was used as the mRNA template and was reverse transcribed into cDNA using iScript Reverse Transcription Supermix (Bio-Rad, cat. #170-8841BUN), which contains a mixture of oligo­(dT) and random primers. Gene expression was quantified by singleplex quantitative real-time PCR (qPCR) using SsoAdvance Universal SYBR Green Supermix (Bio-Rad, cat. #1725274) and PrimePCR SYBR Green Assays for CYP3A4 (Assay ID: qHsaCED0045916) and GAPDH (Assay ID: qHsaCED0038674) as the reference gene. The threshold cycle (*C*_T_) was measured for each target. The mRNA level of CYP3A4 was assessed by relative quantification with GAPDH as the reference gene. The normalized fold induction was calculated by 2^–ΔΔ*CT*^ (Livak) method,^58^ in which DMSO was used as the vehicle control (0%) and rifampicin as the positive control (100%). Each test compound was tested in triplicate at 10 μM. Flumazenil (30 μM) was tested as the negative control. Rifampicin (10 μM) was tested as the positive control.

#### Cytotoxicity Assays

HepG2 cells (1 × 10^4^/well in 25 μL assay media) were added to the wells of white tissue culture-treated 384-well plates (Revvity, cat. #6007680). An Echo 655 Acoustic Liquid Handler was used to dispense 75 nL/well of DMSO or compound dilution, resulting in 0.3% DMSO and the indicated concentrations of chemicals. After 24 h, the CellTiter-Glo Luminescent Cell Viability Assay (Promega, cat. #G7572) and an EnVision microplate reader were used to assess cytotoxicity. DMSO wells with cells served as positive controls, and wells without cells served as negative controls. The percent cell viability for each well was calculated using

$$cell\;viability\;\left(\%\right)=100\times \frac{\left({RLU}_{test\;compound}-{RLU}_{no\;cells}\right)}{\left({RLU}_{DMSO}-{RLU}_{no\;cells}\right)}$$

#### TR-FRET PXR Competitive Binding Assay

The fluorescent PXR ligand BODIPY FL vindoline was synthesized as previously reported.^33^ LanthaScreen Terbium (Tb)-antiglutathione S-transferase (GST, cat. #PV3550) and GST-PXR LBD protein (cat. #PV4841) were purchased from Thermo Fisher Scientific. The TR-FRET PXR competitive binding assay was performed as previously described with modifications.^20,33^ The assay buffer composition was 50 mM Tris (pH 7.5), 0.002% Pluronic F-127, 0.01% bovine serum albumin (BSA), and 0.05 mM dithiothreitol (DTT). BODIPY FL vindoline (150 nM in assay buffer, 10 μL/well) was added into black low-volume 384-well assay plates (Revvity cat. #6008260). Then, stock solutions of test compounds or DMSO (45 nL/well) were dispensed using an Echo 655 Acoustic Liquid Handler (Beckman Coulter Life Sciences). Assay buffer containing Tb-anti-GST (15 nM) and GST-PXR LBD (15 nM) was then added (5 μL/well) to constitute the complete assay mixture (100 nM BODIPY FL vindoline, 5 nM Tb-anti-GST, 5 nM GST-PXR LBD and 0.3% DMSO in a 15 μL final assay volume). DMSO and 10 μM T0901317 were tested as negative and positive controls, respectively. The plates were shaken at 900 rpm (80*g*) on an IKA MTS 2/4 digital microtiter shaker (IKA Works) for 1 min then centrifuged at 1000 rpm (201*g*) for 30 s in an Eppendorf 5810 centrifuge equipped with an A-4–62 swing-bucket rotor. The plates were protected from light exposure and incubated for 90 min at room temperature. After incubation, the TR-FRET signal from each well was collected with a PHERAstar FS Microplate Reader (BMG Labtech) using 340 nm excitation, 520 and 490 nm emissions, a 100 μs delay, and a 200 μs integration time. The measured relative fluorescence units (RFU) were normalized for each well using

$$signal=\frac{RFU\;at\;520\;nm}{RFU\;at\;490\;nm}\times 10^{4}$$

The percentage of PXR LBD binding, demonstrated by the percent FRET signal inhibition in each well, was calculated using

$$PXR\;LBD\;binding\;\left(\%\right)=100\times \left(1-\frac{{signal}_{compound}-{signal}_{T0901317}}{{signal}_{DMSO}-{signal}_{T0901317}}\right)$$

The data were fitted to a sigmoidal dose–response equation to derive IC_50_, which was defined as the concentration of test compound resulting in 50% of FRET signal inhibition. *I*_max_ was defined as the maximum level of FRET signal inhibition achieved by a test compound.

#### Cellular PXR Transactivation Assay

HepG2/C3A cells were obtained from the American Type Culture Collection (ATCC, cat. #CRL-3581) and maintained in Eagle’s Minimum Essential Medium (ATCC, cat. #30-2003) with 10% fetal bovine serum (FBS) (Cytiva, cat. #SH30396.03). Cells were cultured in a humidified atmosphere at 37 °C with 5% CO_2_ and routinely checked for mycoplasma growth using the MycoProbe Mycoplasma Detection Kit (R&D Systems, cat. #CUL001B). Cell counts were obtained by a Countess II Automated Cell Counter using trypan blue staining (Thermo Fisher Scientific). The pcDNA3-FLAG-PXR expression plasmid and pGL3-CYP3A4-luc containing firefly luciferase under the control of a PXR-responsive *CYP3A4* promoter have been previously described.^59,60^ The “assay media” used below was phenol red-free DMEM (Thermo Fisher Scientific, cat. #21063029) supplemented with 5% charcoal/dextran-treated FBS (Cytive, cat. #SH30068.03).

PXR transactivation assays were performed as previously described.^42^ HepG2 cells (7.5 × 10^5^/well in 2 mL culture media) were plated in tissue culture-treated six-well plates (Corning, cat. #353046). The following day, cells were cotransfected with 2 μg/well pGL3-CYP3A4-luc and 100 ng/well pcDNA3-FLAG-PXR using Lipofectamine 3000 (Thermo Fisher Scientific, cat. #L3000015). After 24 h, cells were trypsinized and suspended in assay media, and 1 × 10^4^ cells/well in 25 μL of media were added to white tissue culture-treated 384-well plates. An Echo 655 Acoustic Liquid Handler was used to dispense 75 nL/well of DMSO or stock compounds, resulting in 0.3% DMSO and the indicated concentrations of chemicals. Cells treated with 1 μM T0901317 served as positive controls, and DMSO-treated cells served as negative controls. After incubation for 24 h, a luciferase assay was performed using the steadylite plus Reporter Gene Assay System (Revvity, cat. #6066751) and EnVision microplate reader (Revvity). The measured relative light units (RLU) were normalized for each well using

$$PXR\;activity\;\left(\%\right)=100\times \frac{{RLU}_{compound}-{RLU}_{DMSO}}{{RLU}_{T0901317}-{RLU}_{DMSO}}$$

Antagonist assays were performed with a dose response of rifabutin in the presence of DMSO or 10 μM putative antagonist. The wells contained an additional 0.1% DMSO from the dual addition for a total of 0.4% DMSO. PXR activity was calculated as above using 10 μM rifabutin as the positive control in place of T0901317. All data were fitted to a sigmoidal dose–response equation.

## Supplementary Material





## Notes

Molecular formula strings (CSV)
*M. abscessus* MIC and HepG2 CC_50_ values for compounds **1**–**11**, α12 asymmetry metric calculated from available PXR X-ray crystal structures, average *NPT* ensemble properties for PXR-ligand MD simulations (Tables S1–S3) and structural comparison of the AlphaFold2 loop-filled model and the X-ray cocrystal structure of the PXR LBD with rifampicin bound, docking poses for rifabutin and compounds **1**–**11**, identification of rigid anchor residues in PXR for calculation of the α12 asymmetry metric, dynamics of α12 helix asymmetry in PXR over 200 ns MD simulations for rifabutin, **9** and **7**, fractional occupancy of PXR LBD protein–ligand H-bonds across MD simulations, fractional occupancy of PXR LBD protein–ligand hydrophobic contacts across MD simulations, simulation stability metrics, flexibility of the PXR α12 residues as defined by RMSF (Figures S1–S8) (PDF)

## Abbreviations Used

BSA: bovine serum albumin
CGenFF: CHARMM general force field
CYP: cytochrome P450
DDIs: drug–drug interactions
FBS: fetal bovine serum
GAFF2: general AMBER force field 2
LBD: ligand-binding domain
MD: molecular dynamics
MMFF94×: Merck molecular force field 94×
MIC: minimum inhibitory concentration
MOE: molecular operating environment
NTM: nontuberculous mycobacteria
PCR: polymerase chain reaction
PME: particle mesh Ewald
PXR: pregnane X receptor
RFB: rifabutin
RFU: relative fluorescence units
RLU: relative luminescence units
RMSD: root-mean-square deviation
RMSF: root-mean-square fluctuation
RT-qPCR: reverse transcription quantitative polymerase chain reaction
RXR: retinoid X receptor
SD: standard deviation
SRC-1: steroid receptor coactivator-1
TB: tuberculosis
TR-FRET: time-resolved fluorescence resonance energy transfer
