# Supplementary material for: Rifamycin Structural Modifications Attenuate PXR Binding and CYP3A4 Induction
Source: J Med Chem. 2026 Jun 23;69(13):15858–71. doi: 10.1021/acs.jmedchem.6c00944 (PMC13371085; doi:10.1021/acs.jmedchem.6c00944)
Supplement: Supplementary file 2 [file jm6c00944_si_002.pdf]

Supplementary Information

Rifamycin Structural Modifications Attenuate PXR Binding and CYP3A4 Induction

Amir George,[1,†] Tian Lan,[2,†] Andrew D. Huber,[3,†] Véronique Dartois,[4,5] Thomas Dick,[4,5,6] Taosheng Chen,[3,*] Courtney C. Aldrich,[2,*] Joel S. Freundlich[1,7,*]

1. Department of Pharmacology, Physiology, and Neuroscience, Rutgers University – New Jersey Medical School, Newark, NJ 07103, USA.

2. Department of Medicinal Chemistry, University of Minnesota, Minneapolis, MN 55455, USA.

3. Department of Chemical Biology and Therapeutics, St. Jude Children's Research Hospital, Memphis, TN 38105, USA.

4. Center for Discovery & Innovation, Hackensack Meridian Health, Nutley, NJ 07110, USA.

5. Department of Medical Sciences, Hackensack Meridian School of Medicine, Nutley, NJ 07110, USA.

6. Department of Microbiology and Immunology, Georgetown University, Washington, DC 20057, USA.

7. Division of Infectious Disease, Department of Medicine and the Ruy V. Lourenço Center for the Study of Emerging and Re-emerging Pathogens, Rutgers University - New Jersey Medical School, Newark, NJ 07103, USA.

[†] These authors contributed equally

*Address correspondence to Taosheng Chen (taosheng.chen@stjude.org), Courtney C. Aldrich (aldri015@umn.edu), Joel S. Freundlich (freundjs@rutgers.edu)

Table of contents

Figure S1. Structural comparison of the AlphaFold2 loop-filled model and the X-ray co-crystal structure of the PXR LBD with rifampicin bound.	Page S3
Figure S2. Docking poses for rifabutin and compounds **1 – 11**.	Page S4
Figure S3. Identification of rigid anchor residues in PXR for calculation of the α12 asymmetry metric.	Page S5
Figure S4. Dynamics of α12 helix asymmetry in PXR over 200 ns MD simulations for rifabutin, **9** and **7**.	Page S6
Figure S5. Fractional occupancy of PXR LBD protein-ligand H-bonds across MD simulations.	Page S7
Figure S6. Fractional occupancy of PXR LBD protein-ligand hydrophobic contacts across MD simulations.	Page S8
Figure S7. Simulation stability metrics	Page S9
Figure S8. Flexibility of the PXR α12 residues as defined by RMSF.	Page S10
Table S1. *M. abscessus* MIC and HepG2 CC_{50} values for compounds **1 – 11**.	Page S11
Table S2. α12 asymmetry metric calculated from available PXR X-ray crystal structures.	Page S12
Table S3. Average NPT ensemble properties for PXR-ligand MD simulations.	Page S13
Additional Compound Characterization Data	Page S14

Molecular formula strings (CSV) Please refer to Molecular_formula_strings.CSV

Figure S1. Structural comparison of the AlphaFold2 loop-filled model and the X-ray co-crystal structure of the PXR LBD with rifampicin bound. 1SKX is shown in light gray and the AlphaFold2 model in blue following backbone superimposition. Regions present in the AlphaFold2 model but absent from the X-ray crystal structure are highlighted in orange. The AlphaFold2 model reproduces the overall fold with a backbone Cα RMSD of ~1.2 Å, and differences are largely confined to flexible loop regions lacking X-ray crystallographic electron density. This figure was created with MOE (version 2024.06).

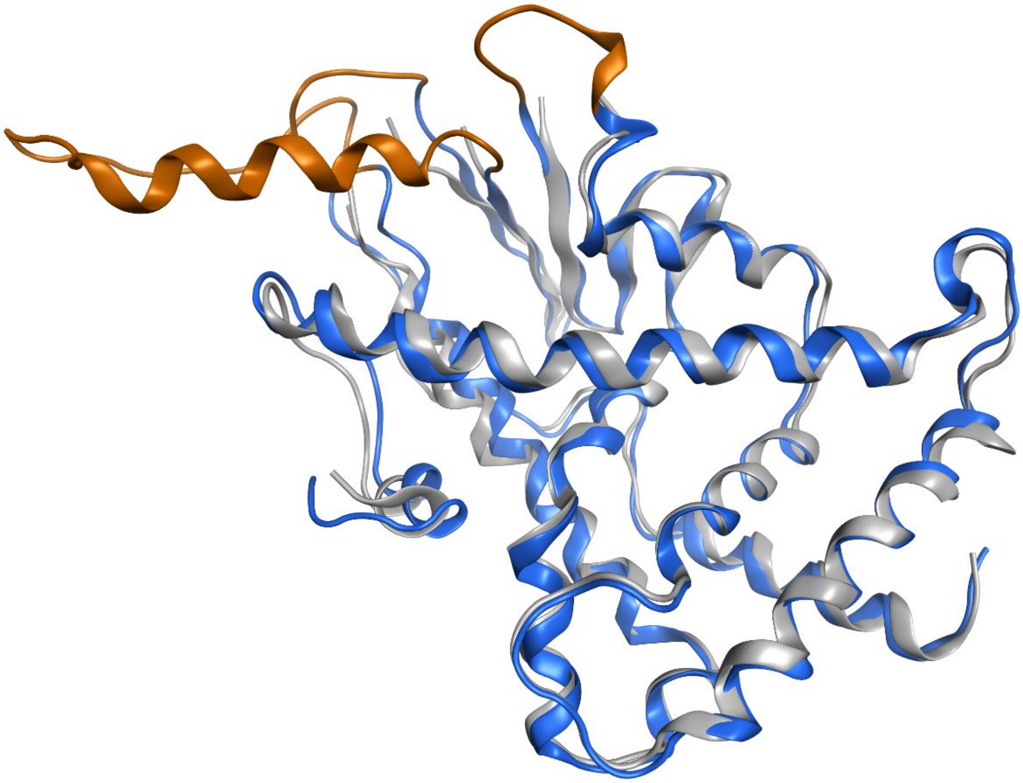

Figure S2. Docking poses for rifabutin and compounds 1 – 11. Docking poses for rifabutin (yellow) and C25-modified analogs (cyan). H-bond (cyan) and hydrophobic (green) interactions with residues Gln285, His407, Arg410, F288 and W299 are shown. This figure was created with MOE (version 2024.06).

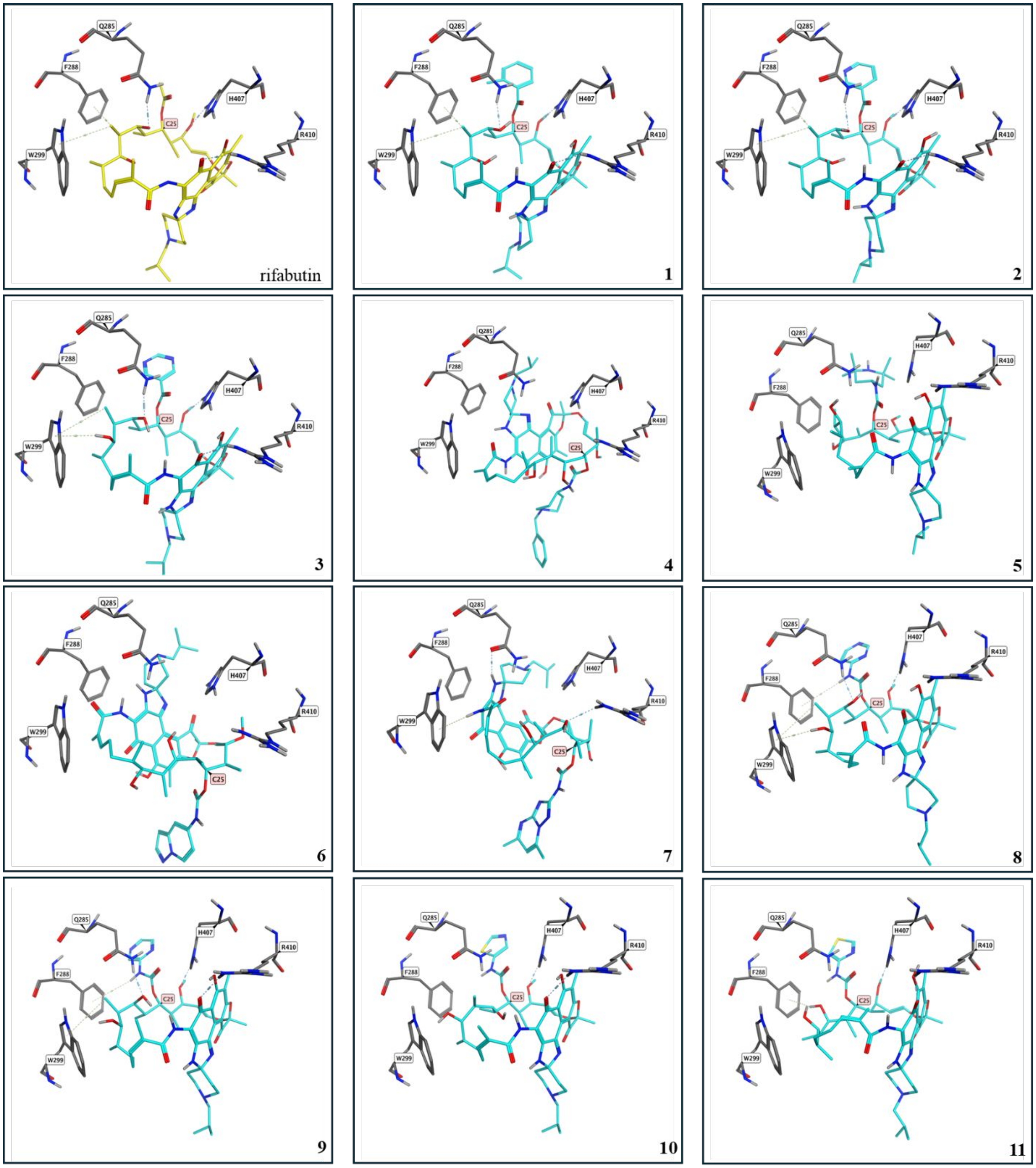

Figure S3. Identification of rigid anchor residues in PXR for calculation of the α12 asymmetry metric. Residue Cα RMSF values were calculated across all simulations, and residues within the lowest 10% were defined as rigid anchors for measuring distances to the α12 helix. Six consistently low-mobility residues were identified and used as reference positions for the asymmetry metric (Leu281, Glu282, Val284, Gln285, Lys286, and Phe288). This figure was created with Python (Version 3.9).

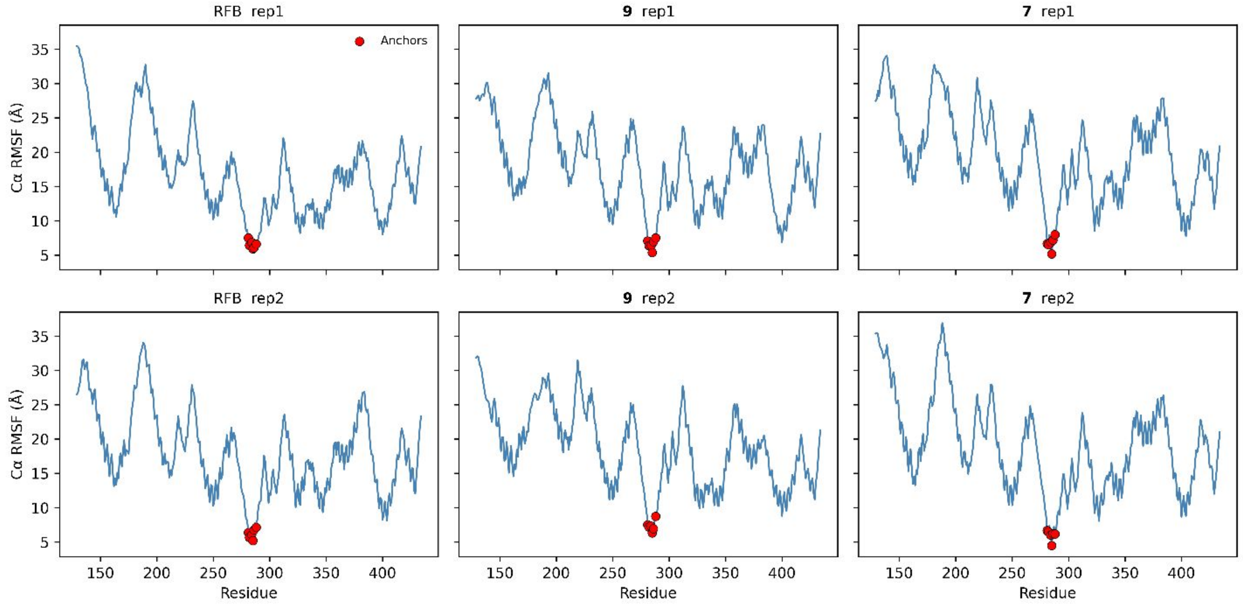

Figure S4. Dynamics of α12 helix asymmetry in PXR over 200 ns MD simulations for rifabutin, 9 and 7. Time evolution of α12 motion over 200 ns MD simulations for rifabutin (top), compound **9** (middle) and compound **7** (bottom) shown for two independent replicates per ligand. The dashed line indicates Δ = 0, corresponding to symmetric positioning of both halves of α12. This figure was created with Python (Version 3.9).

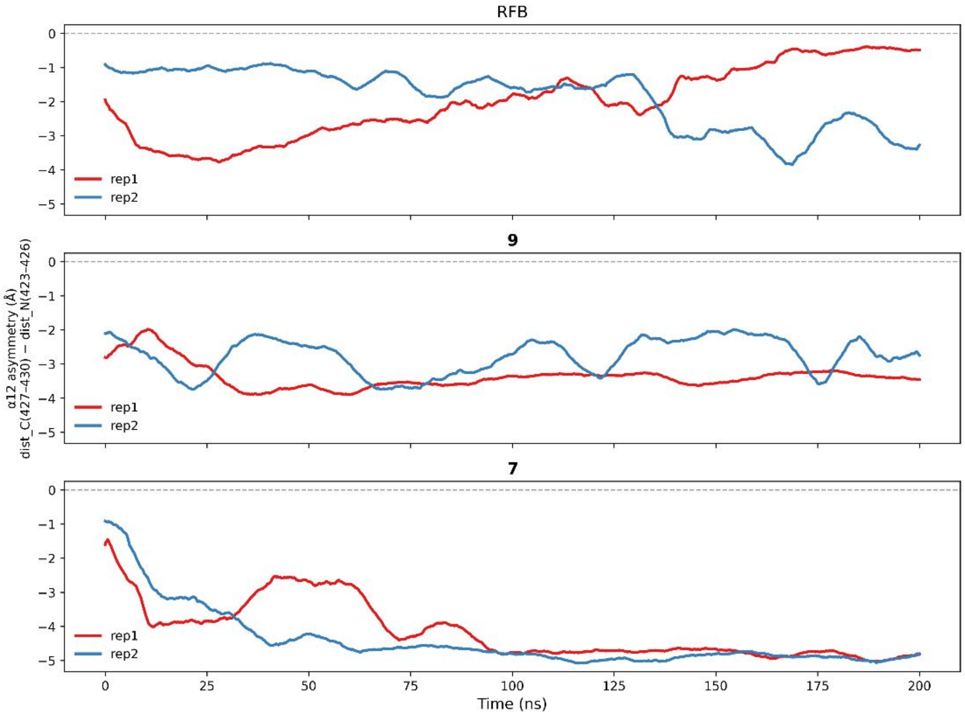

Figure S5. Fractional occupancy of PXR LBD protein-ligand H-bonds across MD simulations. Values represent the average fractional occupancy of each hydrogen bond over the trajectories, averaged across replicate simulations. This figure was created with Python (Version 3.9).

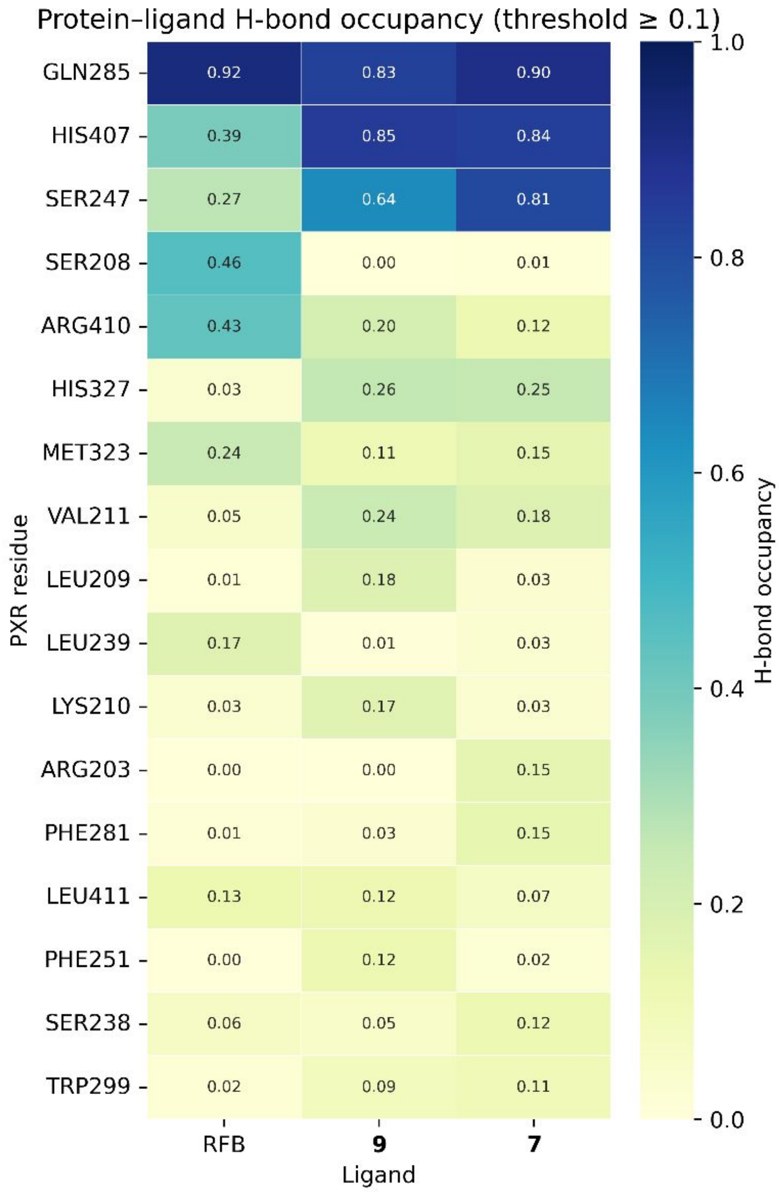

Figure S6. Fractional occupancy of PXR LBD protein-ligand hydrophobic contacts across MD simulations. Values represent the average fractional occupancy of each contact over the trajectories, averaged across replicate simulations. This figure was created with Python (Version 3.9).

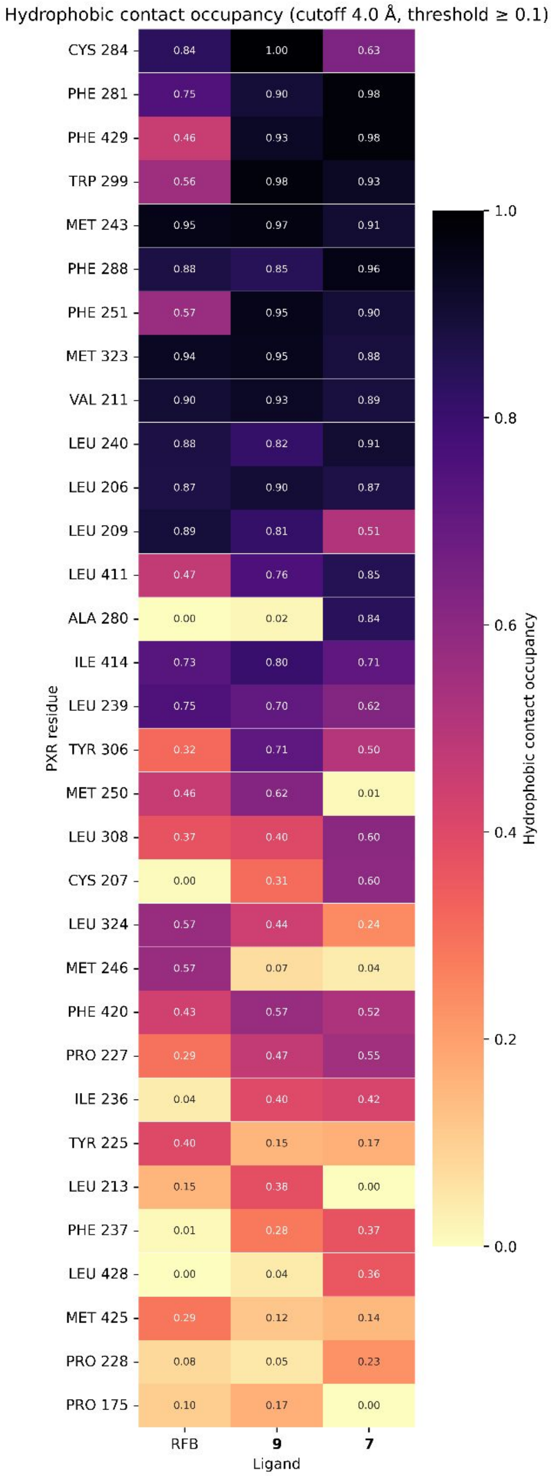

Figure S7. Simulation stability metrics. Time-resolved profiles are shown for three ligands (**RFB**, **9** and **7**), with each column corresponding to one ligand and each row to a different metric. From top to bottom, the metrics are: RMSD (Å), protein radius of gyration, intra-protein H-bonds and ligand RMSD (Å) over 200 ns MD simulations. For each ligand, two independent replicates are overlaid. Raw data are shown as lightly faded traces, with a running mean overlaid to highlight long-timescale trends. This figure was created with Python (Version 3.9).

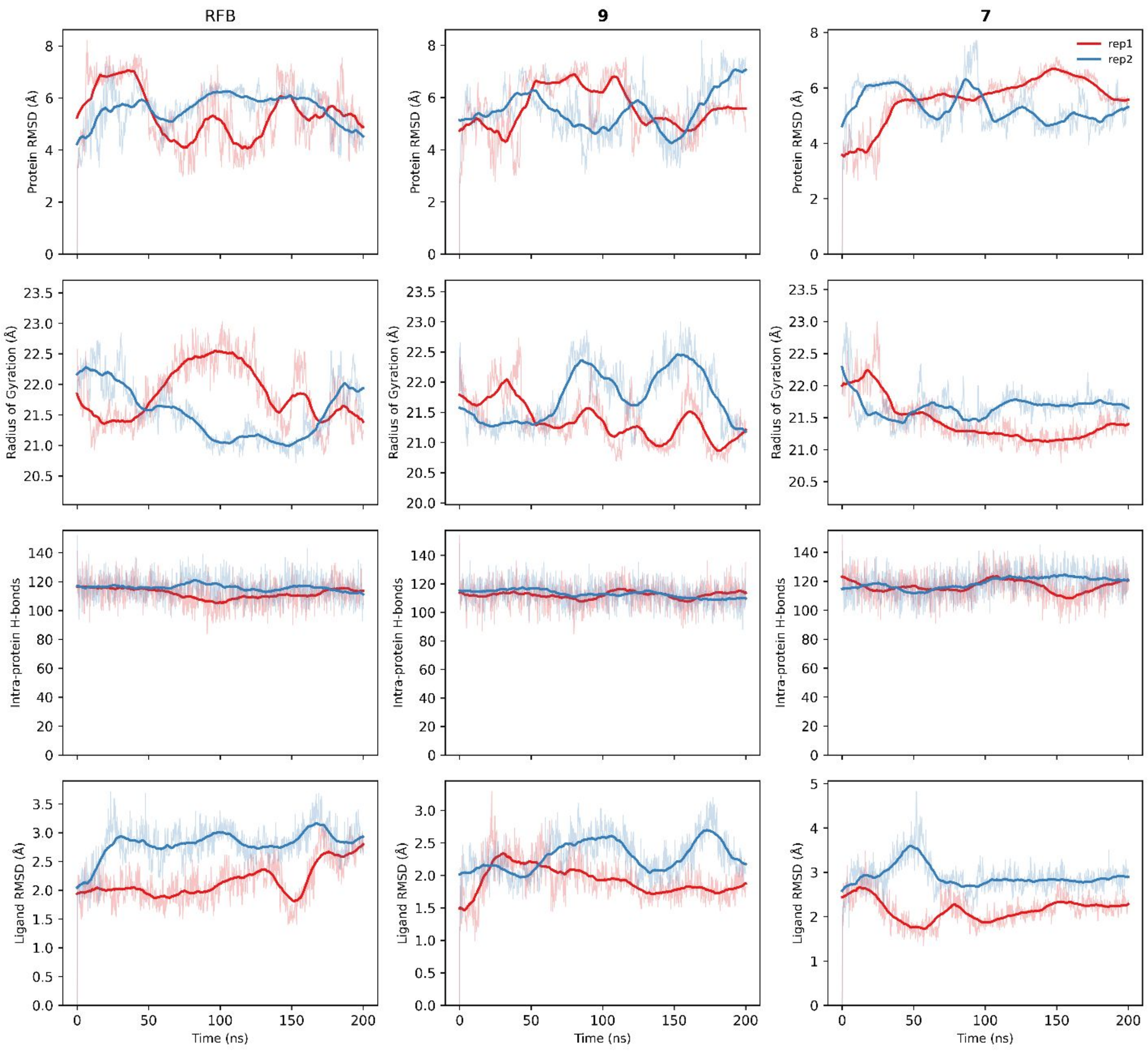

Figure S8. Flexibility of the PXR α12 residues as defined by RMSF. Cα RMSF values for residues 423–434 were calculated across all simulations and replicates. Residues 423–426 defined the N-terminal region of α12, while residues 427–430 defined the C-terminal region used to calculate the α12 asymmetry metric. Residues 432–434 exhibited substantially higher flexibility, consistent with a terminal loop, and were excluded from subsequent asymmetry metric calculations. This figure was created with Python (Version 3.9).

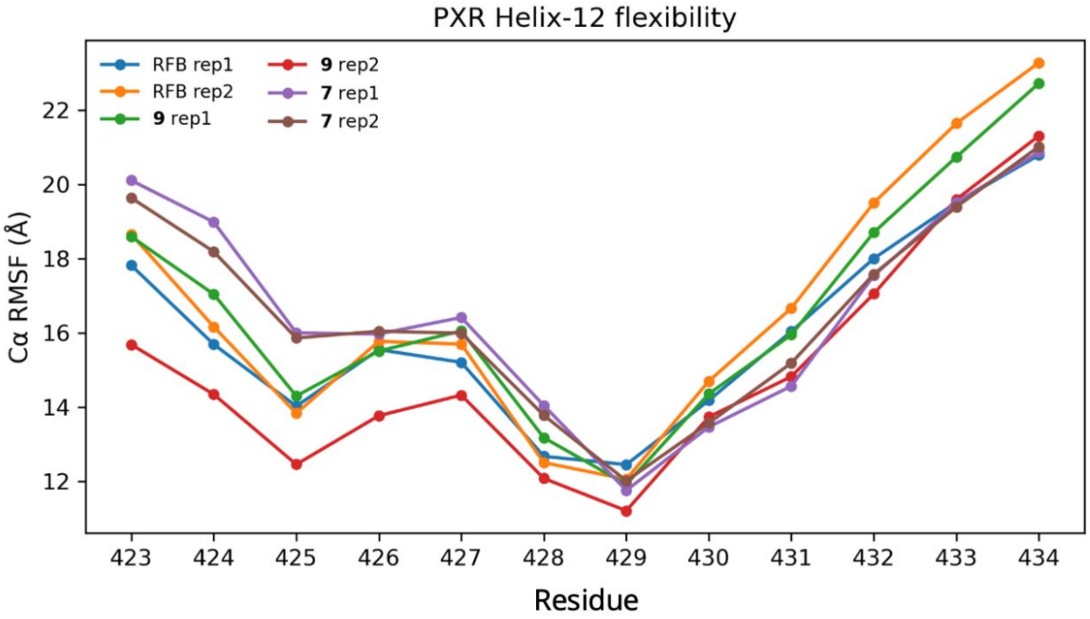

Table S1. ***M. abscessus*** **MIC and HepG2** $\mathbf{CC_{50}}$ **values for compounds 1 – 11.** The UMN numbers were referenced in the cited publications.

**Compound Number (Main Text)**	**Independent UMN Number**	***M. abscessus*** **ATCC 19977 MIC (nM)**	**Reference # (Main Text) for Initial Report of MIC**	**HepG2** $\mathbf{CC_{50}}$ **(µM)[a]**
**1**	UMN16	43	19	8.9 ± 1
**2**	UMN22	24	15	16 ± 0.5
**3**	UMN46	29	15	>30
**4**	UMN15	15	15	2.4 ± 0.3
**5**	UMN32	40	15	5.0 ± 0.3
**6**	UMN123	51	15	3.4 ± 0.6
**7**	UMN137	67	15	>30
**8**	UMN118	32	15	>30
**9**	UMN120	28	15	>30
**10**	UMN132	26	15	11 ± 0.7
**11**	UMN133	46	15	>30

[a] CC_{50} values are reported as mean ± SD for at least four replicates.

Table S2. α12 asymmetry metric calculated from available PXR X-ray crystal structures. The α12 asymmetry metric ($\Delta = d_C - d_N$, in Å) was calculated for a set of PXR LBD X-ray crystal structures representing apo and agonist-bound states using the same anchor residues and α12 split. All structures included had resolutions between 1.5 and 3.0 Å.

PDB ID	Complex	$\Delta = d_C - d_N$
7AXE	Oxadiazon	-0.75
9FZJ	SR12813	-0.97
6HJ2	Dabrafenib	-0.85
7AXF	Pretilachlor	-0.71
7AXI	Estradiol + *cis*-Chlordane	-0.84
7AXK	Estradiol + Endosulfan	-0.71
1M13	Hyperforin	-0.84
1NRL	SR12813 + SRC-1	-1.15
4NY9	BMS-817399	-1.04
7AXA	Clotrimazole	-0.62
4J5W	Apo-PXR/RXRα LBD heterotetramer	-0.99
7AX8	Apo	-0.77

Table S3. Average NPT ensemble properties for PXR-ligand MD simulations. Reported values correspond to the mean ± standard deviation computed over the NPT production phase for each ligand and replicate. These values were used to ensure that the system remained stable prior to production MD.

Ligand	Replicate	Pressure (bar)	Density (kg/m³)	Volume (nm³)	Box X (nm)	Box Y (nm)	Box Z (nm)
RFB	1	-15.49 ± 87.54	1015.76 ± 3.92	1389.63 ± 5.46	12.526 ± 0.016	12.526 ± 0.016	8.857 ± 0.012
RFB	2	-13.81 ± 87.94	1016.31 ± 3.97	1388.88 ± 5.53	12.524 ± 0.017	12.524 ± 0.017	8.855 ± 0.012
**9**	1	-6.56 ± 85.14	1016.66 ± 4.12	1388.44 ± 5.72	12.522 ± 0.017	12.522 ± 0.017	8.855 ± 0.012
**9**	2	-3.66 ± 95.22	1016.16 ± 4.15	1389.12 ± 5.77	12.524 ± 0.017	12.524 ± 0.017	8.856 ± 0.012
**7**	1	-23.02 ± 89.41	1016.19 ± 4.08	1389.23 ± 5.68	12.525 ± 0.017	12.525 ± 0.017	8.856 ± 0.012
**7**	2	8.82 ± 102.31	1016.62 ± 4.12	1388.64 ± 5.73	12.523 ± 0.017	12.523 ± 0.017	8.855 ± 0.012

Additional Compound Characterization Data

As noted in the text, the molecules described in this work have either been published previously or are submitted for publication elsewhere. We include representative LC traces.

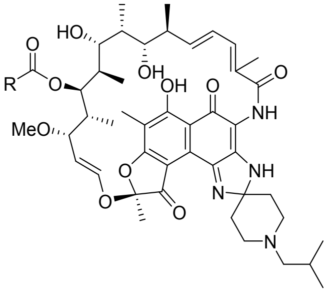

Superscript nd	LC trace
**1**<br>**R =** 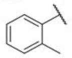	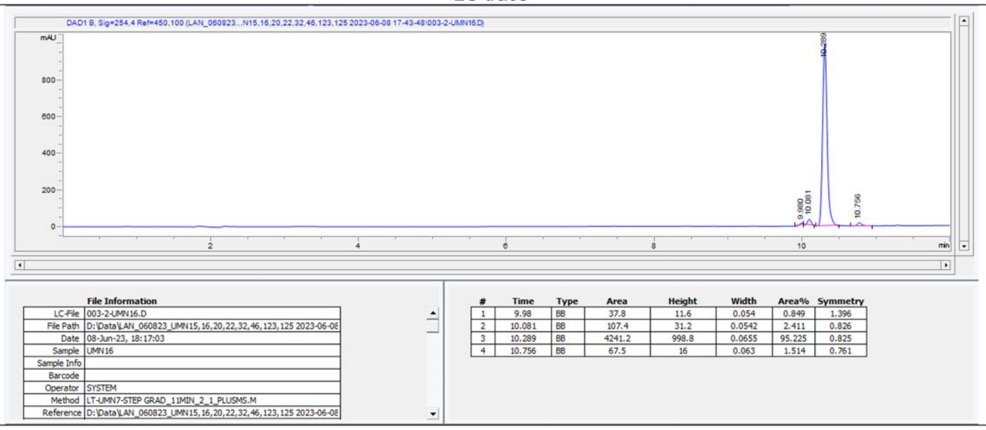

2

R =

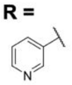

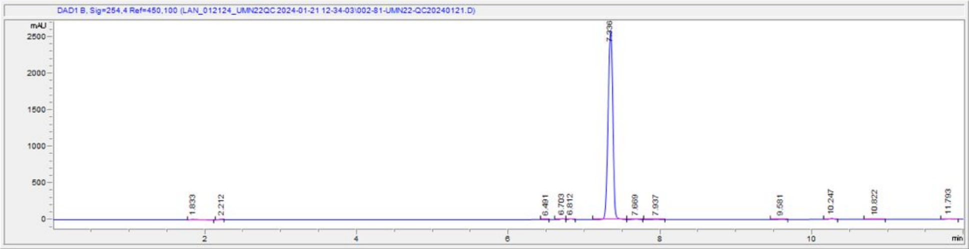

File Information

LC-File	002-81-UMN22-QC20240121.D
File Path	D:\Data\LAN_012124_UMN22QC 2024-01-21 12-34-03
Date	21-Jan-24, 12:51:09
Sample	UMN22-QC20240121
Sample Info	
Barcode	
Operator	SYSTEM
Method	LT-UMN7-STEP GRAD_11MIN_2_1_PLUSMS.M
Reference	D:\Data\LAN_012124_UMN22QC 2024-01-21 12-34-03\001-91-blar
Analysis Time	12 min

#	Time	Type	Area	Height	Width	Area%	Symmetry
1	1.833	BB	54.2	6.6	0.1213	0.470	0.289
2	2.212	BB	13.3	3.7	0.0599	0.115	1.706
3	6.491	BB	6	2.1	0.0478	0.052	1.206
4	6.703	BB	34.1	10.8	0.0508	0.295	1.063
5	6.812	BB	28.1	9	0.0505	0.243	0.874
6	7.336	BB	11281.6	2580.9	0.071	97.737	1.149
7	7.669	BB	24.1	5.3	0.0707	0.209	0.992
8	7.937	BB	18.2	3.4	0.079	0.158	1.434
9	9.581	BB	12.7	2.1	0.0965	0.110	1.095
10	10.247	BB	49.2	13.7	0.0559	0.426	0.942

3

R =

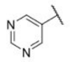

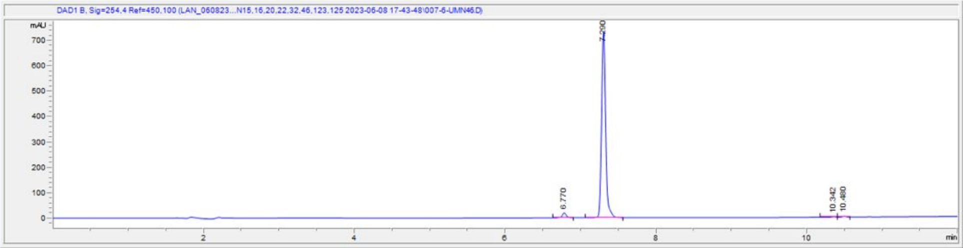

File Information

LC-File	007-6-UMN46.D
File Path	D:\Data\LAN_060823_UMN15,16,20,22,32,46,123,125 2023-06-08
Date	08-Jun-23, 19:20:42
Sample	UMN46
Sample Info	
Barcode	
Operator	SYSTEM
Method	LT-UMN7-STEP GRAD_11MIN_2_1_PLUSMS.M
Reference	D:\Data\LAN_060823_UMN15,16,20,22,32,46,123,125 2023-06-08

#	Time	Type	Area	Height	Width	Area%	Symmetry
1	6.77	BB	72.5	18.8	0.059	2.551	1.011
2	7.29	BB	2750	738	0.0575	96.762	0.855
3	10.342	BB	7.7	1.9	0.0633	0.270	1.273
4	10.48	BB	11.8	3.3	0.0577	0.417	0.904

6

R =

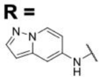

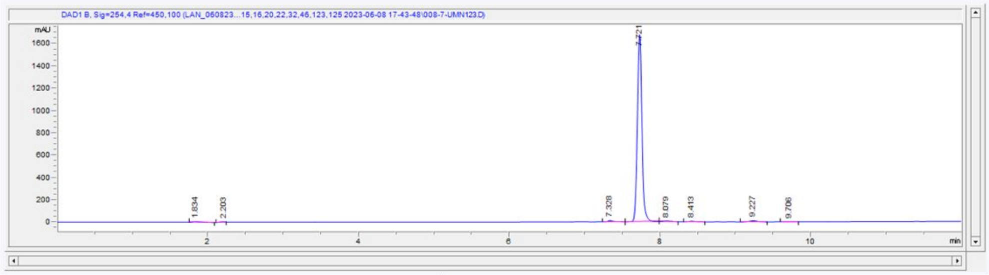

File Information

LC-File	008-7-UMN123.D
File Path	D:\Data\LAN_060823_UMN15,16,20,22,32,46,123,125 2023-06-08
Date	08-Jun-23, 19:36:36
Sample	UMN123
Sample Info	
Barcode	
Operator	SYSTEM
Method	LT-UMN7-STEP GRAD_11MIN_2_1_PLUSMS.M
Reference	D:\Data\LAN_060823_UMN15,16,20,22,32,46,123,125 2023-06-08

#	Time	Type	Area	Height	Width	Area%	Symmetry
1	1.834	BB	41.5	5.2	0.1134	0.566	0.392
2	2.203	BB	15.9	3.6	0.074	0.217	2.221
3	7.328	BB	61	12	0.0732	0.834	0.518
4	7.721	BB	7055.7	1675.7	0.0651	96.398	0.922
5	8.079	BB	38.8	6.8	0.0847	0.530	0.605
6	8.413	BB	29.9	5.2	0.0887	0.408	0.738
7	9.227	BB	70.3	9.9	0.1106	0.961	0.893
8	9.706	BB	6.3	1.2	0.0829	0.086	0.891

9

R =

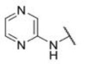

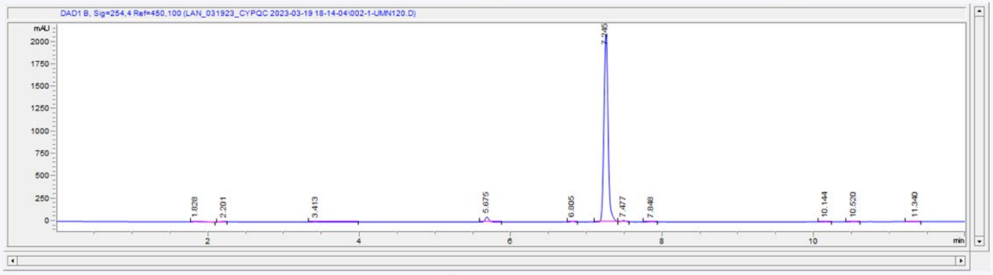

File Information

LC-File	002-1-UMN120.D
File Path	D:\Data\LAN_031923_CYPQC 2023-03-19 18-14-04
Date	19-Mar-23, 18:31:13
Sample	UMN120
Sample Info	
Barcode	
Operator	SYSTEM
Method	LT-UMN7-STEP GRAD_11MIN_2_1_PLUSMS.M
Reference	D:\Data\LAN_031923_CYPQC 2023-03-19 18-14-04\001-91-blank.D

#	Time	Type	Area	Height	Width	Area%	Symmetry
1	1.828	BB	55.2	6.5	0.1207	0.670	0.259
2	2.201	BB	20.2	4.5	0.0741	0.245	1.966
3	3.413	BB	11	2.2	0.0755	0.133	0.494
4	5.675	BB	197.6	55.6	0.0554	2.397	0.831
5	6.805	BB	22.8	7.1	0.0493	0.277	0.916
6	7.245	BB	7845.5	2115.6	0.0593	95.164	0.902
7	7.477	BB	44.1	12.6	0.057	0.535	0.828
8	7.848	BB	24.4	4.8	0.0811	0.296	1.023
9	10.144	BB	8.1	2.1	0.0616	0.098	0.902

10

R =

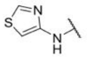

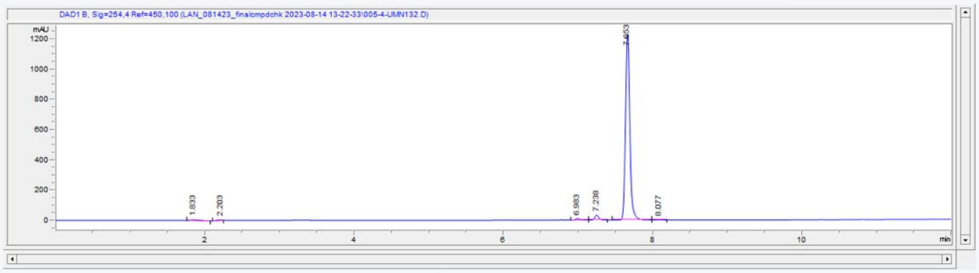

File Information

LC-File	005-4-UMN132.D
File Path	D:\Data\LAN_081423_finalcmpdchk 2023-08-14 13-22-33
Date	14-Aug-23, 14:27:32
Sample	UMN132
Sample Info	
Barcode	
Operator	SYSTEM
Method	LT-UMN7-STEP GRAD_11MIN_2_1_PLUSMS.M
Reference	D:\Data\LAN_081423_finalcmpdchk 2023-08-14 13-22-33\001-91-b

#	Time	Type	Area	Height	Width	Area%	Symmetry
1	1.833	BB	41.7	5.3	0.1115	0.797	0.391
2	2.203	BB	17.2	3.6	0.0779	0.329	2.373
3	6.983	BB	40.8	10.3	0.0603	0.780	0.812
4	7.238	BB	123.8	33.1	0.0576	2.366	0.815
5	7.653	BB	4999.9	1231.5	0.0634	95.572	0.869
6	8.077	BB	8.1	1.8	0.071	0.155	0.847
